# Beyond negative valence: 2-week administration of a serotonergic antidepressant enhances both reward and effort learning signals

**DOI:** 10.1371/journal.pbio.2000756

**Published:** 2017-02-16

**Authors:** Jacqueline Scholl, Nils Kolling, Natalie Nelissen, Michael Browning, Matthew F. S. Rushworth, Catherine J. Harmer

**Affiliations:** 1 Department of Experimental Psychology, University of Oxford, Oxford, United Kingdom; 2 Department of Psychiatry, University of Oxford, Oxford, United Kingdom; 3 Oxford Centre for Human Brain Activity, Department of Psychiatry, University of Oxford, Oxford, United Kingdom; 4 Oxford Centre for Functional MRI of the Brain (FMRIB), Nuffield Department of Clinical Neurosciences, University of Oxford, Oxford, United Kingdom; University of Cambridge, UNITED KINGDOM

## Abstract

To make good decisions, humans need to learn about and integrate different sources of appetitive and aversive information. While serotonin has been linked to value-based decision-making, its role in learning is less clear, with acute manipulations often producing inconsistent results. Here, we show that when the effects of a selective serotonin reuptake inhibitor (SSRI, citalopram) are studied over longer timescales, learning is robustly improved. We measured brain activity with functional magnetic resonance imaging (fMRI) in volunteers as they performed a concurrent appetitive (money) and aversive (effort) learning task. We found that 2 weeks of citalopram enhanced reward and effort learning signals in a widespread network of brain regions, including ventromedial prefrontal and anterior cingulate cortex. At a behavioral level, this was accompanied by more robust reward learning. This suggests that serotonin can modulate the ability to learn via a mechanism that is independent of stimulus valence. Such effects may partly underlie SSRIs’ impact in treating psychological illnesses. Our results highlight both a specific function in learning for serotonin and the importance of studying its role across longer timescales.

## Author summary

Drugs acting on the neurotransmitter serotonin in the brain are commonly prescribed to treat depression, but we still lack a complete understanding of their effects on the brain and behavior. We do, however, know that patients who suffer from depression learn about the links between their choices and pleasant and unpleasant outcomes in a different manner than healthy controls. Neural markers of learning are also weakened in depressed people. Here, we looked at the effects of a short-term course (2 weeks) of a serotonergic antidepressant on brain and behavior in healthy volunteers while they learnt to predict what consequences their choices had in a simple computer task. We found that the antidepressant increased how strongly brain areas concerned with predictions of pleasant and unpleasant consequences became active during learning of the task. At the same time, participants who had taken the antidepressant also performed better on the task. Our results suggest that serotonergic drugs might exert their beneficial clinical effects by changing how the brain learns.

## Introduction

To make good decisions in complex environments, humans and animals need to learn about and integrate different sources of information, such as the good and bad aspects of the outcomes of choices. The neurotransmitter serotonin has been implicated in value-based choice and assumed to play a role in value learning, but even basic aspects of its function in such learning remain contested.

Serotonin has recently been implicated in simple aspects of reward-guided learning and decision-making. For example, when serotonergic neuron activity is recorded on a millisecond timescale or levels are manipulated acutely, serotonin has been found to code information about different aspects of good/appetitive [1–4] or bad/aversive [5,6] outcomes or to relate to avoidance behaviors [7,8]. On this basis, it has been suggested that it has a role in learning [9,10]. However, other studies have found no effects on learning [3,11–14] or have not dissociated learning from altered responsiveness to the valence of the reinforcing events themselves [15]. Thus, there is still no clear understanding of serotonin’s role in value learning. Here, we propose that this gap can be bridged by examining the effects of serotonin on value learning over a different timescale.

From animal studies, it is known that serotonin not only transfers information on millisecond timescales but also acts over protracted timescales of days and weeks. In fact, at the neuronal level, prolonged increases in serotonin over such timescales lead to changes in plasticity [16,17]. Increasing serotonin levels over several weeks, for example, by administering selective serotonin reuptake inhibitors (SSRIs), can reintroduce juvenile-like plasticity in the visual cortex [18] and the limbic system [19] in animals. Moreover, such effects are, respectively, linked to improvements in learning about visual stimuli and fear extinction. Interestingly, the timeframe of plasticity observed in animals is very similar to the timeframe for antidepressants to take effect in patients, so it may be particularly revealing to study serotonin’s neural and behavioral effects during learning at this timescale.

We therefore examined here whether prolonged increases in serotonin affect learning about appetitive (monetary reward) and aversive outcomes (investment of effort) independently of any effects such a manipulation may have on the coding of stimulus outcome valence per se. In other words, we examined whether serotonin increase has any effect on how we learn from pleasant or unpleasant outcomes as well as any direct impact on responsivity to the pleasant and unpleasant events **per se**. Using effort as the unpleasant dimension appeared particularly relevant in the context of serotonin’s role in treating clinical depression, in which motivation deficits are observed [20,21]. We recruited 29 human participants, who received 20 mg/d of the SSRI citalopram for 2 weeks or placebo (in a double-blind design), a similar dosage to that used clinically. Repeated administrations of SSRIs for this period of time have been shown to increase serotonin levels in nonhuman primates [22] and related markers of serotonin levels in humans [23–25]. A two-week administration schedule was chosen, as this is similar to the timeframe of appearance of early clinical effects of SSRIs in depression [26], of behavioral change in mild-stress animal models of depression [17], and of changes in neural plasticity in animals [17]. Participants subsequently performed a learning task while we measured their brain activity using functional magnetic resonance imaging (fMRI). In the task [27], participants concurrently learned about the changing values of two stimuli between which they had to choose on each trial. The value of each stimulus was determined by a pleasant (amount of monetary reward) and an unpleasant (amount of effort) dimension (we ensured that participants perceived the reward dimension as rewarding and the effort as aversive, see S1 Text #2 “Task description—training”). Our task allowed us to measure the neural and behavioral effects SSRIs might have on learning signals for pleasant and/or unpleasant information. It also made it possible to dissociate the effect SSRIs had on responses to receipt of positive and negative outcomes per se as opposed to the effect **on learning about** positive or negative outcomes. We hypothesized that if SSRIs affected learning independently of coding of valence, it should strengthen neural learning signals for both dimensions similarly. We found that SSRIs led to stronger reward and effort-related learning signals (i.e., reward and effort prediction errors [RPEs and EPEs]—the neural responses to the differences between the received and the expected outcomes) in a widespread network of brain areas coding value information. At the same time, however, activity related simply to the receipt of reward/effort outcomes per se was unaffected. This suggests that prolonged SSRI administration directly influences learning signals in humans over and above any effect it has on signaling pleasant or unpleasant outcomes per se. Not only were neural learning signals stronger but, at a behavioral level, we found that reward-related learning in complex environments was improved.

## Results

### Task and study design

This study investigated how neural and behavioral measures of reward and effort learning were modulated by repeated administration of an SSRI when both dimensions needed to be learned concurrently and could potentially interfere with one another.

To address this, healthy human participants (for details, see S1 Text #1 “Participants”) performed a previously established multidimensional learning task [27,28], while we measured their brain activity using fMRI. Participants were randomly assigned to 2 weeks of a clinical dose of the SSRI citalopram (20 mg/d, *n* = 15) or placebo (*n* = 14). Participants did not differ in any sociodemographic measures, and citalopram did not lead to any changes (baseline versus after 2 weeks of treatment) for any self-reported scores of depression, anxiety, positive or negative affect, or mood state (S1 Table).

**Fig 1:**
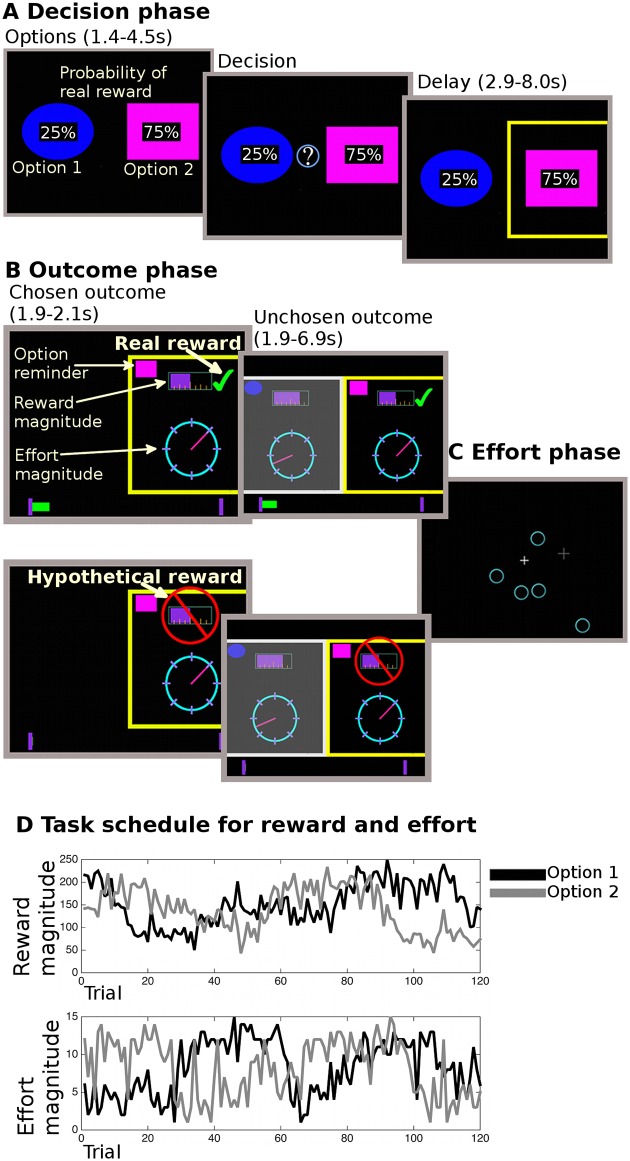
Task description. In the decision phase (A), participants were shown two options (i.e., choices) overlaid with a cue (percentage number) informing them of the probability of receiving a real (rather than hypothetical) reward for each choice. They could only decide after an initial monitoring phase (1.4–4.5 s). The chosen option was then highlighted for 2.9–8s. In the following outcome phase (B), participants saw the outcome for the chosen option first (1.9–2.1 s). The reward magnitude was shown as a purple bar (top of the screen); the effort magnitude was indicated through the position of a dial on a circle. Whether they received a reward (i.e., the trial’s reward type) was indicated by a green tick mark (real reward, top display) or a red crossed out sign over the reward magnitude (hypothetical reward, bottom display). If a reward was real, the reward was also added to a status bar at the bottom of the screen, which tracked rewards over the course of the experiment. A reminder of which option had been chosen was shown at the top of the screen. Then, the reward and effort magnitudes were shown for the unchosen option (1.9–6.9 s). Finally, participants performed the effort phase (C), in which participants needed to exert a sustained effort by selecting circles that appeared on the screen using a trackball mouse. The number of targets was equivalent to the chosen effort outcome. Participants had to perform the effort phase on every trial independently of whether the reward was real or hypothetical. Participants successfully completed the effort phase on almost every trial. Participants performed a fixed number of 120 trials per session (thus, selecting options with less effort did not have a lower opportunity cost, i.e., it did not allow participants to perform more trials for more overall reward). An example schedule is shown in (D), with both the reward and effort magnitude values of the two options. Figure is adapted from Figure 1 [27].

The task [27] is described more extensively in the supporting methods (S1 Text, #2 “Task description”). In short, in the task, participants repeatedly chose between the same two options, aiming to choose the options maximizing their monetary gain and minimizing the effort they needed to exert to obtain the reward (Fig 1). When making their decisions (Fig 1A), they therefore had to take into account the independent reward and effort magnitudes associated with each of the two options, which they had to learn from experience across trials. These magnitudes slowly varied over the course of the experiment (Fig 1D). At the time of the decision, participants were additionally shown on the screen the randomly drawn probability of how likely each option was to lead to a real or hypothetical reward outcome (the probability determined what we later refer to, for the sake of brevity, as the “reward type” of a choice). If an option led to what we called a real reward outcome, participants received the reward magnitude points as monetary pay-off for the experiment; if an option led to a hypothetical reward outcome, participants were only shown how much money they could have won for this choice, but were not awarded it as monetary pay-off on this particular occasion. As these reward probabilities were randomly drawn on each trial, the reward type (real versus hypothetical) of one trial should not influence participants’ decisions on the next trial. However, we have shown previously [27] that reward type on one trial nevertheless biased participants’ behavior on the next trial in this learning task.

**Fig 2:**
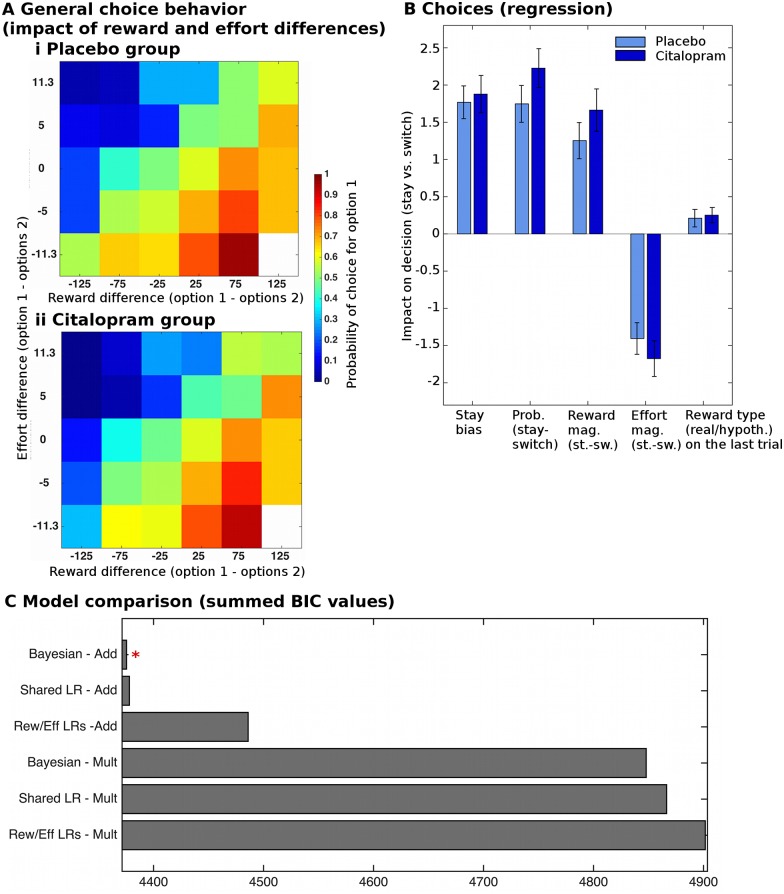
Task validation and model comparison. (A) The choices of participants in both groups (i, ii), between option one and option two, were guided by the learnt reward and effort differences between the options (estimated from a Bayesian model). They were more likely to choose the option with higher reward and lower effort magnitudes. (B) Regression analysis (bGLM1) predicting whether participants selected the same option again as on the last trial (“stay”) or selected the alternative option (“switch”). Participants took all relevant features of the task into account: they were more likely to choose options that had a higher displayed probability, higher learnt reward, and lower effort magnitudes (all *p* < 10^−8^; no group differences, all *p* > 0.2; omnibus ANOVA including regression weights for probability, learnt reward and effort also revealed no group difference: F(1,27) = 2.3, *p* = 0.14). Participants were also more likely to choose an option again if they had received a real reward on the last trial (t(28) = 3.04, *p* = 0.005). There was no difference between the groups in the overall amount of money earned. (C) Model comparison using summed Bayesian Information Criterion (BIC) values revealed that models in which choice utility was computed as a linear sum (i.e., reward + probability − effort, “Add”) provided a far better fit to the data than models computing choice utility multiplicatively (i.e., reward x probability—effort, “Mult”). Of these models, a Bayesian model (no free parameters for learning rate, reward/effort predictions are instead derived using Bayes’ rule) provided the best fit to the data (“Bayesian—Add”: BIC = 4375), closely followed by a model in which there was one free and shared parameter for the reward and effort learning rate (“Shared learning rate—Add”: BIC = 4378). The regressors for learnt reward and effort magnitudes used in the behavioral and neural analyses derived from “Bayesian—Add” were highly correlated with regressors derived from “Shared learning rate − Add” (r > 0.99). Error bars are standard error of the mean. Data for individual participants can be found in S1 Data.

After each choice, participants saw the outcome of their choice (Fig 1B). At this time point, participants could learn about the reward and effort magnitudes of the two options. In other words, they could update their effort/reward expectation if it was violated, i.e., if the current trial’s outcome deviated from their prior expectation. Note that a numeric expression of how much one’s prediction deviated from the actual outcome and therefore should be changed is called the prediction error (PE), see below. This task was designed to be challenging for participants: ideally, they should simultaneously learn about both the reward and effort magnitude associations of the options regardless of whether rewards were real or hypothetical. Thus, optimal learning needed to represent all these components separately to prevent them interfering with one another. In constructing the task schedule (example in Fig 1D), we ensured that all factors of interest, e.g., the behavioral and neural measures of learning about reward and effort, varied independently from each other. This ensured, as in other studies [3,27,29,30], that their independent effects on behavior and neural activity could be determined (S4 and S11 Figs). After careful training, participants showed a good understanding of the task and good performance (Fig 2A and 2B and S1 Text #2 “Task description—Training”).

### fMRI

In order to quantify value learning, simple computational models have been proposed [31]. A key component of these models are PEs, the difference between the actual and the expected outcome, or, in other words, how much better (or worse) than expected the outcome is. Such PEs then drive learning, i.e., they lead to changes in predictions for the next occasion that a choice can be taken. It has been shown previously that neural correlates of PEs can be found in different areas of the human brain using fMRI and that they relate to behavioral markers of learning [32–37]. We therefore tested whether citalopram affected PEs as the neural substrate of learning. Later, we examined how these changes translated into behavioral changes. Neural correlates might be more proximal to the molecular level action of citalopram than behavioral measures, which are the integrated (and binarized) outputs of many different brain processes. If citalopram increased synaptic plasticity and therefore induced learning-related changes in neural activity, then this should manifest in increased PE signals. We found this to be the case for both reward and effort learning.

### Increased serotonin enhances reward and effort prediction errors (EPEs)

We measured neural learning signals (PEs) at the time of the outcome. The regressors for this analysis were derived from a Bayesian learning model ([27] and S1 Text #3 “Bayesian model”), which provided a good fit to our data (Fig 2A–2C). Using a fitted Rescorla—Wagner reinforcement learning model instead produced the same neural and behavioral results.

**Fig 3:**
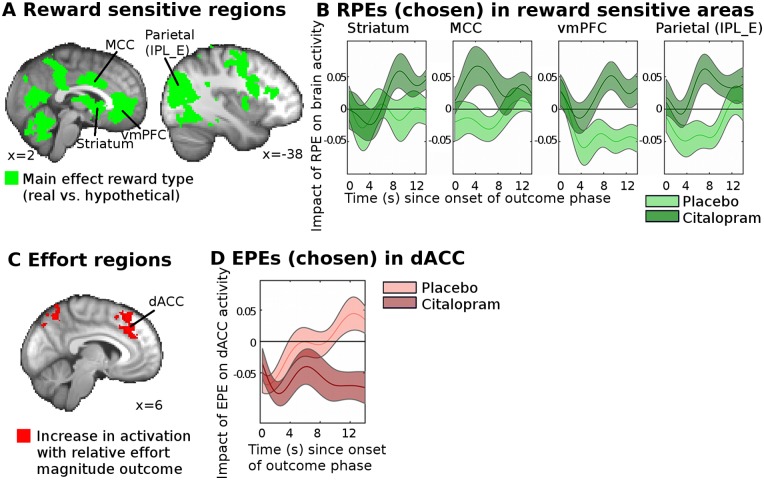
Citalopram leads to a widespread increase in reward and effort learning signals. (A) We identified ROIs that were sensitive to reward type (i.e., whether reward was really received or only hypothetical, analysis fGLM1) at whole-brain level (*p* < 0.05). Abbreviations: ventral striatum (striatum), midcingulate cortex (mCC), ventromedial prefrontal cortex (vmPFC), parietal cortex (Parietal, IPL_E, [38]). (B) Shows the time course of the regression coefficients in these ROIs for the RPE of the chosen option on the neural BOLD signal for the placebo (light green) and the citalopram (dark green) groups (analysis fGLM2). Citalopram increased the RPE signal across all ROIs (ANOVA, group difference across all areas, i.e., difference in the mean value across all four areas: F(1,27) = 9.48, *p* = 0.005). This effect was driven by the citalopram group showing an activation across all four areas (ANOVA including all four areas, citalopram group only, activation across all areas: F(1,14) = 7.66, *p* = 0.015), while the placebo group did not show any change in BOLD (ANOVA including all four areas, placebo group only, no activation or deactivation across all areas: F(1,13) = 2.20, *p* = 0.16). Next, we performed similar analyses for the effort dimension. (C) We first identified dorsal anterior cingulate cortex (dACC) and other areas (S2 Table and S2 Fig) as being sensitive to the relative effort outcome. (D) Shows the regression coefficient for the EPE of the chosen option on the neural BOLD signal in dACC for the placebo (light red) and the citalopram (dark red) groups. Again, citalopram significantly enhanced the EPE signal (t(27) = 3.01, *p* = 0.006; significance threshold for Bonferroni correction for six brain areas is *p* < 0.008), making it more negative like the EPE signal in other brain areas (S2 Fig). The pattern of group differences for RPEs and EPEs across the whole brain (at a reduced statistical threshold) is shown in S3 Fig. Brain maps and data for individual participants can be found in S2 and S3 Data.

To test whether citalopram affected neural correlates of reward PEs (RPEs), we first selected regions of interest (ROIs) that were sensitive to the receipt of reward (i.e., whether reward was real or hypothetical, analysis fGLM1, Fig 3A, table of coordinates in S2 Table, results cluster-corrected at *p* < 0.05, voxel inclusion threshold: z > 2.3) and that had previously been implicated in the processing of rewards or learning [33,34]. These regions included striatum and ventromedial prefrontal cortex (vmPFC). Using the averaged BOLD data from these regions, we then tested whether citalopram affected the neural RPE signals (i.e., the difference between the received and the expected reward magnitude, independent of whether reward was real or hypothetical). This analysis was thus statistically independent of any analyses used to establish the ROIs in the first place. We found (Fig 3B, analysis fGLM2) that in those ROIs, citalopram strongly enhanced the neural correlates of RPEs for the chosen option (an ANOVA revealed significant group differences across all areas, i.e., difference in the mean value across all areas: F(1,27) = 9.21, *p* = 0.005; an additional analysis [fGLM2_reduced_] not controlling for reward/effort outcomes produced the same result: F(1,27) = 7.3, *p* = 0.012; *t* tests for each area individually are as follows: striatum: t(27) = −1.74, *p* = 0.093; mid cingulate cortex: t(20.24) = −2.12; *p* = 0.048, vmPFC: t(27) = −2.88; *p* = 0.008, parietal cortex: t(27) = −2.64, *p* = 0.014). Supplementary analyses confirmed that this result was robust to different RPE modeling choices (S1 Fig).

We next performed an analogous analysis to find neural correlates of EPEs. First, we identified effort outcome—related brain areas by finding areas that became more active when the chosen option was associated with more effort than the option that was unchosen (we refer to this contrast as the relative effort outcome contrast; Fig 3C, S2 Fig, S2 Table, analysis fGLM1). As the neural correlates of effort processing have received comparatively less attention than those related to reward processing, there were no strong a priori hypotheses about which regions might carry EPEs. However, despite the relative absence of specific information about EPEs, dorsal anterior cingulate cortex (dACC) has been consistently linked to effort processing in both animals and humans [39–44]. We therefore tested for EPEs in all effort-sensitive regions, although prior work suggested that dACC should be a focus of particular interest. Again, note that the EPE contrast is independent from the contrast used to establish the ROIs in the first place. In all ROIs, we examined whether citalopram increased neural correlates of EPEs. In all areas, higher EPEs led to a decrease in activity (S2 Fig). An ANOVA across these areas revealed that in some areas EPEs were stronger (more negative) in the citalopram group (interaction effect group x area: F(5,135) = 2.45, *p* = 0.037; main effect of group: F(1,27) = 1.43, *p* = 0.24, analysis fGLM2; an additional analysis [fGLM2_reduced_] not controlling for reward/effort outcomes suggests a group difference main effect across all tested brain areas: F(1,27) = 4.8, *p* = 0.037, interaction effect group x area: F(3.8,101.5) = 3.34, *p* = 0.015). This effect was particularly striking in dACC (between-subject *t* test: t(27) = 3.01, *p* = 0.0056, significance threshold of Bonferroni correction for six brain areas: *p* < 0.008; Fig 3D). No significant differences were found in the other areas (S2 Fig).

In summary, our fMRI results showed strong evidence that repeated administration of citalopram increased neural correlates of learning signals for both reward and effort. This is in agreement with what would be predicted if an increase in synaptic plasticity in value learning—related brain regions is induced by repeated citalopram administration.

### Neural learning effects are not the result of increased outcome processing

As control analyses, we tested whether changes in learning with repeated SSRI administration might be secondary to increases in the coding of appetitive or aversive outcomes per se. This is based on theories that suggest that serotonin is involved in the coding of the valence of outcomes [8,45]. In our paradigm, PEs were sufficiently decorrelated from the outcomes themselves (S4 Fig) so that the impact of SSRIs on both could be investigated in the same analysis. This was possible because participants needed to learn reward and effort magnitudes rather than probabilities (the reward probabilities associated with the options did not need to be learned because this information was explicitly cued and provided to the participants on every trial; Fig 1).

**Fig 4:**
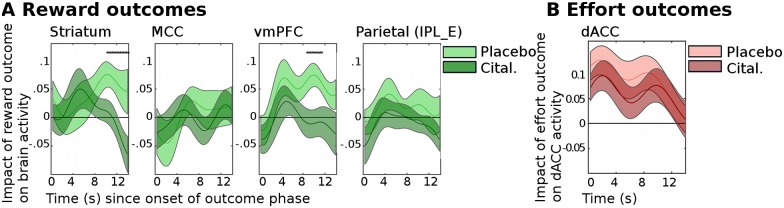
Citalopram does not increase neural signals for reward or effort outcomes. (A) Shows the time course of the regression coefficients for the relative (chosen minus unchosen option) reward magnitude outcomes on brain activity (analysis fGLM2) for the placebo (light green) and the citalopram (dark green) groups. We found that citalopram did not increase the relative reward magnitude outcome signal (ANOVA, testing for a main effect of group across all areas: F(1,27) = 1.19, *p* = 0.29). On the contrary, a more lenient time point—by—time point *t* test analysis of the time courses revealed that in striatum and vmPFC, citalopram, in fact, decreased the relative reward magnitude outcome signal late in the outcome phase (*p < 0.05 for time point—by—time point two-sided *t* tests). (B) Similarly, for dACC, citalopram did not increase the coding of the relative effort outcome signal (t(27) = 0.65, *p* = 0.52). Abbreviations: ventral striatum (striatum), midcingulate cortex (mCC), ventromedial prefrontal cortex (vmPFC), parietal cortex (Parietal, IPL_E [38]), dorsal anterior cingulate cortex (dACC). Data for individual participants can be found in S4 Data.

Therefore, we tested whether citalopram also affected the reward magnitude outcome signals (analysis fGLM2, regressor of reward magnitude outcome). We found that citalopram did not increase reward magnitude outcome signals in the ROIs that had shown increased RPEs with citalopram (Fig 4A; ANOVA, group difference across all areas: F(1,27) = 1.19, *p* = 0.29) or on a whole-brain level. On the contrary, there was some evidence for reward outcome signals to be reduced in striatum and vmPFC by citalopram in a relatively late period during the processing of the outcomes. In other words, although there was no significant difference between the groups in the hemodynamically convolved signals time-locked to the onset of the outcome phase, there were statistical differences in the time course of the BOLD signal late during the outcome phase when using a more lenient statistical approach that did not correct for multiple comparisons (Fig 4A).

Similarly, we next tested (analysis fGLM2) whether citalopram affected the coding of effort magnitude outcomes in ROIs sensitive to effort as identified above. Again, this was not the case in an ANOVA across all six ROIs (ANOVA, testing for a group difference across all areas: F(1,27) = 0.29, *p* = 0.60), nor was it the case more specifically in dACC, in which citalopram had increased EPEs (Fig 4B; *t* test comparing the effort outcome signals across the two groups: t(27) = 0.65, *p* = 0.52) or on a whole-brain level.

In a further control analysis, we tested whether citalopram affected the BOLD response in general (rather than specifically increasing RPE/EPE signals). This was not the case (S5 Fig).

Together, our fMRI results suggest that citalopram enhanced neural learning signals independently of any increases to reward or effort outcome sensitivity per se.

### Citalopram makes reward learning more robust against interference

Having found that citalopram increased neural learning signals, we next tested whether it also affected behavioral markers of learning. The analogous behavioral test is to measure the impact of PEs on behavior, as has been done previously [29,37,46,47] (learning can also be measured using alternative methods, S7 Fig). In short, this approach assesses to what extent PEs on one trial have an impact on participants’ choices on the next trial. For example, one would expect that if there is a positive RPE on one trial (i.e., the option is better than expected) then this should make participants more likely to select the option again on the next trial. The approach is thus very related to the neural regression analyses, in which we measured the impact of PEs on brain activity, rather than choices: a regressor that is “chosen” in the outcome phase of one trial (fMRI), is a regressor favoring “stay” (i.e., choosing again the same option) on the next trial (behavioral analysis).

Improved neural PE signals could translate into different kinds of behavioral learning improvements. They might result in a general overall improvement in using PEs to drive behavior. This was not the case (all *p* > 0.26, S7 Fig). This was probably because participants in the placebo group were already generally very good at learning, making it difficult to measure further general improvements. However, even if it is the case that a general, overall improvement is not observable, there may still be evidence of improvements if we focus on situations in which learning is particularly challenging. In the present task, participants needed to simultaneously learn about reward and effort; furthermore, reward was only hypothetical (rather than real) on some trials. Learning in these trials is particularly challenging. Optimally, participants should learn similarly from both real and hypothetical reward outcomes; even if the latter have less intrinsic value, they should still be equally informative for learning. Similarly, learning about reward magnitudes should be independent from learning about effort magnitudes. We therefore hypothesized more specifically that learning might be subject to some degree of interference from irrelevant factors and that this might be remedied by citalopram: learning about one dimension (e.g., reward) might be interfered with to some degree by the absence of real reward experience (i.e., when rewards were only hypothetical) and/or having to learn about the other dimension simultaneously. For example, a surprisingly high effort outcome might attract processing resources so much that reward learning is impaired. Citalopram’s enhancing effect on neural learning signals **in general** might then manifest in the behavior as more robust learning **specifically** in the face of interference.

**Fig 5:**
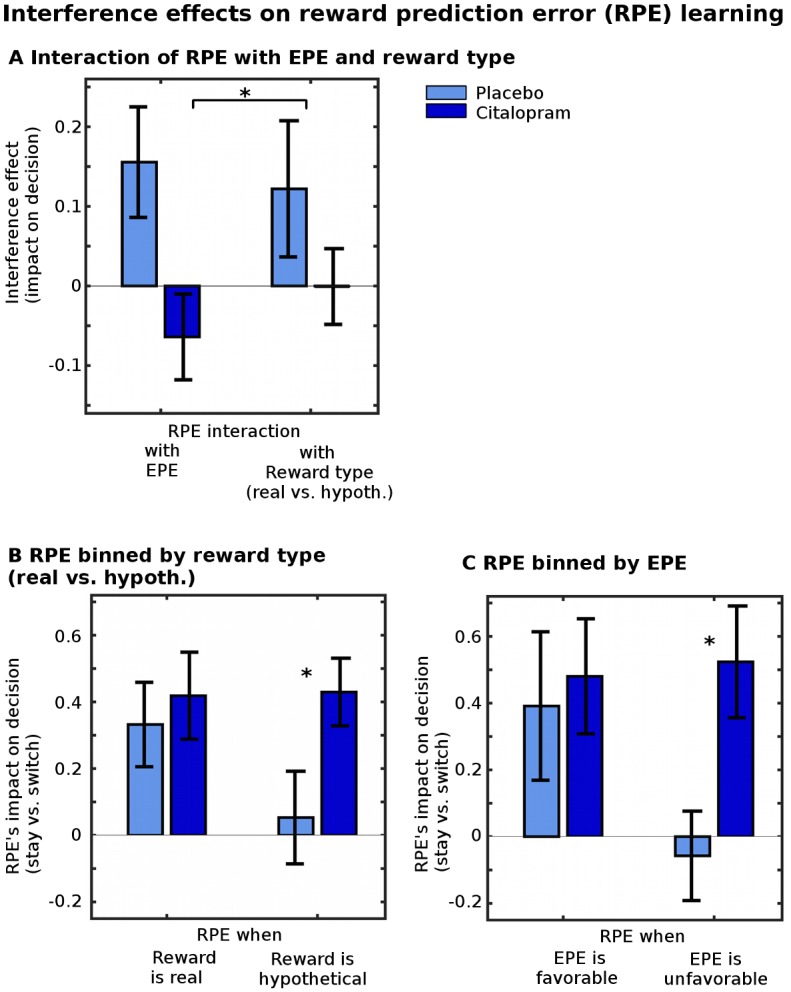
Citalopram protects reward learning from interference. (A) A regression analysis (bGLM2) assessed interference in reward learning (impact of relative RPEs) by effort learning (relative EPEs) and reward type (receiving a real or only a hypothetical reward). Larger regression weights indicate larger interference. While the placebo group’s learning was affected by interfering factors (ANOVA, testing the average size of the two interference factors against zero in the placebo group: F(1,13) = 5.39, *p* = 0.033), this was remedied by citalopram (ANOVA, testing the average size of the two interference factors against zero in the citalopram group: F(1,14) = 1.04, *p* = 0.32; ANOVA, testing whether the two groups differed in the average size of the two interference factors: F(1,27) = 7.00, *p* = 0.013). This effect can be illustrated more directly by comparing how much participants could take RPEs into account for making decisions on the next trial when there was interference or when there was not (analyses bGLM3a and b). (B) When reward was real, the two groups did not differ in how well they could use RPEs (t(27) = −0.47, *p* = 0.64). However, when reward was only hypothetical, the citalopram group was better at using RPEs (t(27) = −2.21, *p* = 0.036). (C) Similarly, when EPEs were favorable, the two groups did not differ in how well they could use RPEs (t(27) = −0.32, *p* = 0.75), but when EPEs were unfavorable, the citalopram group was better at using RPEs (t(27) = −2.69, *p* = 0.012). Error bars show standard error of the mean, *p < 0.05. Data for individual participants can be found in S5 Data.

To test the impact of citalopram on such learning, we performed a regression analysis (bGLM2) that assessed how much participants’ decisions on each trial to “stay” (i.e., to select again the same option as on the last trial) or to “switch” to the alternative option took the PE into account differently in the face of interfering factors (either the fact that the RPE involved a reward that was just hypothetical or the fact that the RPE occurred in the context of a high EPE). In this regression, interference was measured as an interaction between the interfering factors (reward type [i.e., real reward versus hypothetical reward] and EPEs) and the RPEs (see Methods for list of additional confound regressors included). Significant positive interaction terms between RPE and reward type (real versus hypothetical) or EPEs would then mean that participants were not as efficient at using the RPEs when reward was hypothetical compared to real or when effort was surprisingly high. However, if the interaction terms were not different from zero, then it would mean that participants were equally efficient at using RPEs whether rewards were real or hypothetical and regardless of whether the EPE was high. The analysis showed (Fig 5A) that the reward learning of participants in the placebo group was more subject to interference than that of participants taking citalopram (the placebo participants had larger regression weights for the interaction terms: ANOVA, measuring the average interference effect across both interaction term regression weights, comparing the two groups: F(1,27) = 7.00, *p* = 0.013). This effect is illustrated in Fig 5B and 5C (analyses bGLM3a and b): When there was no interference, because rewards were real and EPEs were low, the two groups did not differ in how much they could use RPEs on one trial for making decisions on the next trial (between-subject *t* tests for group differences: when reward was real [bGLM3a]: t(27) = −0.47, *p* = 0.64; when EPEs were favorable [bGLM3b]: t(27) = −0.32, *p* = 0.75). However, when there was potential for interference, because rewards were hypothetical or EPEs were surprisingly high, only the citalopram group still used RPEs for decisions on the next trial (between-subject *t* tests for group differences: when reward was hypothetical: t(27) = −2.21, *p* = 0.036; when EPEs were unfavorable: t(27) = −2.69, *p* = 0.012; one-sample *t* tests within each group testing whether RPEs significantly affected decisions: when reward was hypothetical: placebo: t(13) = 0.38, *p* = 0.71; citalopram: t(14) = 4.23, *p* = 0.001; when EPEs were unfavorable: placebo: t(13) = −0.43, *p* = 0.68; citalopram: t(14) = 3.13, *p* = 0.007).

This effect was specific to learning about the reward dimension (S8 Fig). This was potentially so because effort was always real (it had to be exerted on every trial) and, therefore, potentially easier to learn about. We also note that while the behavioral learning effects were selective for situations of interference, neural learning signal improvements were always present rather than specific to situations of interference (S9 Fig).

As a last behavioral finding, we also noted that citalopram did not affect how participants exerted effort, nor did it disrupt how motivation affected effort exertion (S10 Fig). Thus, just as citalopram did not affect neural responses to reward and effort outcomes per se, so it had no impact on the effect of reward or effort outcomes per se on different behavioral measures.

In summary, we found that citalopram not only enhanced PE coding at a neural level, but it also enhanced the impact of PEs at a behavioral level. Citalopram made RPE-based learning more robust against interference.

## Discussion

This study examined the role of serotonin in value learning by looking at the effects of a repeated administration of the selective serotonin reuptake inhibitor (SSRI) citalopram on reward and effort learning in human participants. Participants performed a multidimensional learning task in which it was necessary to learn about both reward and effort. Neurally, we found that citalopram increased learning signals, i.e., PEs, for both reward and effort. RPEs were increased in a widespread network of brain regions, including vmPFC. At the same time, EPEs were increased in dACC. This increase in learning signals occurred in the absence of increases to the overall outcome signals for reward or effort. Behaviorally, we found that citalopram made reward learning more robust or resilient to negative interference.

### SSRIs enhance neural measures of learning

Citalopram enhanced neural learning signals for both pleasant and unpleasant outcomes across many brain areas. This general and widespread effect is in agreement with a general increase in learning and plasticity after repeated SSRI administration [48], rather than a specific effect on only either appetitive or aversive learning.

One brain area that we identified as having increased RPE signals was the vmPFC. This area has repeatedly been identified as being involved with reward-guided reversal learning [49]. Furthermore, it has been shown that depletion of serotonin levels in adjacent prefrontal areas impaired reversal learning in marmosets [50–52]. While our results further support the claim that serotonin affects the role of vmPFC in reversal learning, we also note that our results point to an effect of SSRIs on learning that is not selective to vmPFC, as we found changes in the RPE signals in a wide range of areas. This is unsurprising given the systemic administration of citalopram used here.

EPEs were also enhanced, particularly in dACC. Although less is known about the neural mechanisms of effort processing, compared to reward processing, the dACC has been linked to aspects of effort processing in both animal models and humans [28,39–43]. Arguably, in the present paradigm, reward learning was more challenging than the more straightforward effort learning; while some level of effort exertion was constantly required on every trial, knowledge of the changes in reward magnitudes that the participants learned had to be integrated with information about reward probabilities presented on each trial, and the potentially distracting fact of whether or not a reward was real or hypothetical had to be ignored. The more widespread impact of the SSRI we studied on RPEs than EPEs may, therefore, simply reflect the more challenging nature of reward learning in this task.

Our results thus suggest an important role for serotonin in the control of value-guided learning from both pleasant and unpleasant outcomes. This is in agreement with findings that in patients with obsessive-compulsive disorder (OCD), who were given SSRIs as part of their treatment, performance in a learning task with monetary wins and losses was improved [53]. These learning effects may relate to changes in synaptic plasticity. SSRIs have been shown to lead to plasticity changes in the brain when administered repeatedly, and other studies have found that serotonin levels naturally increase in situations in which new learning occurs [16]. Furthermore, when SSRIs are administered for a prolonged time in animal models, there have been reports of an impact on basic forms of learning, such as visual adaptation and fear extinction [16,18,19]. This has been linked to changes in synaptic plasticity [16], synapse remodeling [17], and neurogenesis in the hippocampus [54] in animals. In other words, when serotonin levels are changed for a prolonged time, a series of adaptive downstream changes occur, which ultimately lead to increased learning and plasticity. This is in contrast to studies reporting shorter timescale serotonin manipulations in humans, for example, through tryptophan depletion, that have found no effect on reward learning [3,11–15,55–58], but see also [10] and [9]. While the present results reveal that sustained serotonin manipulation has a causal influence on the neural correlates of RPEs and EPEs and on learning, it was not possible to identify the various downstream changes that mediated the impact of serotonin, and it is possible that these included other neurotransmitter changes [59] or might produce other independent effects on behavior beyond improvements in learning. For example, studies have found SSRI administration to reduce GABA levels [18], to make certain forms of long-term potentiation easier to induce [18,19,60], to increase markers of LTP [16], to increase neurogenesis, and to change the morphology of neural dendrites [17]. Further work in animals will be needed to elucidate how these mechanisms relate to value learning.

### Learning signal increases are not secondary to increases to reward/effort receipt coding

Importantly, beyond demonstrating increased neural learning signals, our neural data also allowed us to rule out the possibility that the effects of SSRIs on learning were secondary to an effect on the coding of positive or negative outcomes per se. If this were the case, we would have expected SSRIs to increase signals for reward and effort receipts (“outcomes”) at the same time as increasing learning signals. However, we instead found that SSRIs did not increase neural reward or effort outcome signals, and, if anything, at a lower statistical threshold, we found that in vmPFC and striatum, reward outcome signals were decreased. This is similar to previous studies that found decreases in brain activity to rewarding stimuli with prolonged SSRI administration [61–63]. This is also in agreement with optogenetic studies that failed to find evidence for serotonergic activity per se being reinforcing or aversive [1,4].

While our longer-term manipulation of serotonin did not reveal effects on the processing of aversive outcomes that could explain the learning effects, we note that previous studies, particularly looking at the function of serotonin at shorter timescales or through genetic variations in serotonin transporter polymorphisms, have found evidence for a role of serotonin in (aversive) outcome processing or inhibition [2,6,7,10,14,15,55,58,64–68]. Rather, what our study suggests is that, over the longer term, serotonin additionally plays an independent role in value-guided learning by modulating learning capability directly, rather than just as an indirect consequence of any impact it has on appetitive or aversive outcome signals over shorter timescales.

### SSRIs enhance behavioral measures of learning and plasticity

Beyond changes to neural markers of learning, we also found, at a behavioral level that repeated administration of SSRIs increased learning. A priori, there are several ways in which improved neural learning signals could translate into improved learning behaviorally. Firstly, in classic theories [31], learning is about establishing expectation about mean magnitudes or probabilities. In such a scenario, learning can be influenced by a general change in the speed of learning this expectation, i.e., by changing the learning rate. Secondly, learning can also be improved by changing the precision of the representation of the learnt information, i.e., by increasing the signal-to-noise ratio. Importantly, in the second scenario, better learning can mean being able to use the learnt information more consistently in situations in which learning is particularly challenging, for example, because of interference from other outcome value dimensions. Neurally, a more precise estimate of learning would be reflected in stronger PE signals. Our findings thus align best with the second scenario: neural PEs were increased by the SSRI, but learning speed per se was not changed. Additionally, the SSRI had a protective effect on learning in challenging situations in which negative interference would normally have drawn resources away from the processing of key reward-related contingencies. Neurophysiologically, increased signal-to-noise could be achieved by SSRIs changing the properties of individual synapses, the number of synapses in a state that allows learning [17], or by allowing better integration between the predictions and the outcomes. Such cellular changes could either increase the signal itself or reduce the noise—both possibilities would improve how the information for learning is represented in the brain.

### Clinical relevance

The results of our study might shed light onto the mechanism by which SSRIs work as treatments for psychological illnesses, such as depression. Early clinical effects have been reported with the same dosage and duration at which we administered citalopram to our participants [26,48]. In fact, our results might suggest that SSRIs exert part of their clinical effect by enhancing how well patients can learn about positive relationships in complex environments even in the presence of negative interfering information, which otherwise could prevent such learning. This effect may occur in addition to, or even underlie, previous reports that SSRIs shift the processing of social cues so that they are perceived as more positive [69]. In fact, these two effects, relating to learning and attentional biases, might interact with each other, resulting in patients perceiving the world as more positive and learning more reliably about positive aspects of the world. In this context, it is noteworthy that our behavioral results are in agreement with other studies that found that changes in serotonin levels affect how much negative stimuli can bias behavior [70,71].

### Limitations

In the fMRI data, we noted that while the citalopram group showed strong overall RPEs, the placebo group did not. This is in contrast to previous reports of PEs in healthy participants performing probabilistic reward tasks. There were, however, some differences in our task and analyses: our participants learned about changing reward magnitudes rather than changing reward probabilities, and our analysis carefully dissociated RPE responses from responses to reward delivery per se. Nevertheless, it is important not to over-interpret the lack of strong RPEs in the placebo group as evidence of absence; it is quite possible that the placebo groups’ brains carried overall RPE signals that were simply below our threshold for detection. However, and most importantly, we can conclude from our data that the citalopram group had stronger overall RPE signals than the placebo group.

Furthermore, while the neural PE effects we found were not reflected in general changes in all aspects of PE-based behavioral learning, we did observe some more specific changes in aspects of PE-based behavioral learning. Namely, we observed improved RPE-based learning in situations of interference. This may be so because the serotonergic manipulation acted directly on the brain, but those neural changes only impacted behavior in certain situations in which learning was particularly challenging (because of interfering factors) and, therefore, most likely to be subject to disruption. We do not want to rule out the possibility of finding more general effects in future studies using larger participant samples or other tests of RPE-based learning.

### Conclusion

We found that repeated administration of an SSRI increased neural PE signals during reward and effort learning. Concomitantly, behavioral measures of reward learning in the face of negative interference were improved. Thus, prolonged administration of SSRIs can strengthen learning signals for both appetitive and aversive outcomes in a manner that is consistent with previous demonstrations of the impact of serotonin manipulation on neural plasticity. These results are also of clinical relevance, supporting theories that SSRIs’ treatment effects on, for example, depression may be related to increases in neural plasticity and learning.

## Methods

### Ethics statement

Participants gave written informed consent to take part in the study, which was approved by the NRES Committee South Central—Portsmouth (12/SC/0276).

### fMRI analyses

All analyses were performed in FSL, Matlab, and SPSS. Greenhouse—Geisser corrections for violations of sphericity and nonparametric tests were used where appropriate.

### MRI data acquisition and preprocessing

Structural MRI and fMRI measurements were taken using a Siemens 3 Tesla MRI scanner (see S1 Text #5, “MRI” and [27]). In short, we used a Deichmann echo-planar imaging sequence [72]. We used FMRIB’s Software Library (FSL) [73] for image preprocessing and analysis. All main effect images shown are cluster-corrected (*p* < 0.05) with the standard voxel inclusion threshold of z = 2.3. We also analyzed data in ROIs, extracted from spheres with a three-voxel (or two, for small brain structures, i.e., striatum) radius, identified in MNI standard space on the basis of orthogonal whole-group contrasts.

### Whole-brain

In the first fMRI analysis, we investigated whether citalopram affected neural learning signals and, more broadly, which brain areas were sensitive to reward and effort information (analysis fGLM1, similar design as previously described [27]). The regressors used in this design were as follows (all correlations between regressors were r < 0.44, S4 Fig): We used three boxcar regressors, indicating the onset and duration of the decision phase (from the beginning of the trial until participants made a choice), the onset and duration of the outcome phase (from the appearance of the chosen outcome until the chosen and the unchosen outcomes disappeared from the screen), and, lastly, the effort exertion phase (from the appearance of the first effort target until participants had removed the last target). We included the following parametric regressors in the decision phase: whether the reward of the chosen option had been real or hypothetical on the last trial, reward and effort magnitude predictions (derived from a Bayesian learning model as described previously [27,74], see S1 Text #3 “Bayesian model” for additional details), and the reward probabilities that were displayed on the screen. In the outcome phase, we included the following parametric regressors: the reward type (real versus hypothetical) delivered for the chosen option, the reward probability for the chosen option, the reward and effort magnitude outcomes for the chosen and the unchosen option, and the RPEs and EPEs for the chosen and the unchosen option. The onset of all regressors for the chosen option was time-locked to the onset of the outcome phase; the onset and duration of the regressors for the unchosen option were time-locked to their display. In each case, separate regressors for the chosen and the unchosen option were used; they were later combined to derive relative (i.e., chosen minus unchosen option) values at the contrast level. In the effort execution phase, we included the clicking rate as a parametric regressor. Finally, we included, as confound regressors, six movement regressors and a regressor indexing when additional visual stimuli were presented to warn participants that they had not clicked the targets on time and that the halfway point of the experiment had been reached. We used FSL’s FLAME [75] to perform higher-level analysis; the two groups were modeled as separate groups with shared variance; outlier de-weighting was used. To identify ROIs (see below and also S1 Text #5 “MRI”), both groups were combined at the contrast level, i.e., we identified areas that showed activations (or deactivations) across both groups.

### Time course analyses

We used analysis fGLM1 to identify regions for time course analyses. Specifically, we used the contrast of reward type to identify ROIs for analyses of RPEs and reward outcomes and we used the contrast of relative effort outcomes to identify ROIs for analyses of EPEs and effort outcomes (Figs 3 and 4). The ROIs were identified on the basis of the peaks of the relevant whole-brain cluster-corrected activations. The aim of theses analyses was to test whether citalopram affected PE and/or outcome signals. We therefore tested whether the two groups differed in reward/effort PEs and in the coding of the reward/effort outcomes (analysis fGLM2). Please note that these analyses were orthogonal to how the ROIs were identified. All time courses were extracted, regressed, and statistically tested as described in Scholl et al. [27] and similar to previous studies [49,76,77] (S6 Fig).

In analysis fGLM2, we included as regressors of interest the RPEs and EPEs (separately for the chosen and the unchosen option) as well as the relative (chosen minus unchosen option) reward and effort magnitude outcomes. As regressors of no interest, we included the reward type (real versus hypothetical) PE (i.e., the reward type of the trial minus the probability of the chosen option that had been shown at the time of choice):

$$Y_{fGLM2}=\;\beta_{0}+\beta_{1}RPE_{chosen}+\beta_{2}RPE_{unchosen}+\beta_{3}EPE_{chosen}+\beta_{4}EPE_{unchosen}+\beta_{5}RewardOutcome_{chosen-unchosen}+\beta_{6}EffortOutcome_{chosen-unchosen}+\beta_{7}RewardTypePE_{chosen}$$

PEs were included as separate regressors for chosen and unchosen options, as previous work [78] suggested that different areas might carry signals for chosen and unchosen PEs. In contrast, chosen and unchosen outcome signals have been found to be present in the same areas with opposite signs [27,49] and were, therefore, included as a relative value regressor. Analysis fGLM2 was time-locked to the onset of the outcome phase. In this, as in all other regressions, all regressors were z-score normalized. We tested whether RPEs, EPEs, reward, or effort outcomes were affected by citalopram using repeated-measures ANOVAs within the sets of ROIs. Significant group differences (omnibus main effects or interactions) were then followed up using *t* tests to assess whether these effects were driven by group differences in specific regions. For example, for EPEs, we performed an ANOVA across the neural data from all ROIs identified in the effort outcomes contrast. Follow-up *t* tests then examined group differences separately in all ROIs. To look at reward and effort PEs, it is also possible to construct a variant of fGLM2 in which no regressors for reward and effort outcomes, or, in other words, fewer nuisance regressors, are included (“fGLM2_reduced_”). Note that in this case, PE regressors are not just the orthogonal component left after parceling out outcomes and may also capture signal variation due to outcomes per se. This is because PEs and outcomes are not completely uncorrelated (S4 Fig).

### Behavioral analyses

#### Task validation

To assess whether participants took all features of the task into account when making their choices, we performed a logistic regression analysis (bGLM1). We predicted participants’ decisions to “stay” (choose the same option again as on the last trial) or “switch” (choose the alternative option compared to the last trial); as regressors we included the relative probabilities (i.e., the probability in favor of the “stay” minus the probability in favor of the “switch” option) shown at the time of choice, the relative learnt reward and effort magnitude predictions (derived from the Bayesian model see S1 Text #3 “Bayesian model”), and the reward type of the previous trial (whether a reward had been real or hypothetical). All regressors were z-score normalized.

$$\begin{array}{l} Y_{bGLM1}=\;\beta_{0}+\beta_{1}RewardProbability_{t}+\beta_{2}RewardMagnitudePrediction_{t}+\beta_{3}EffortMagnitudePrediction_{t}+\beta_{4}RewardType_{t-1} \end{array}$$

### Model fitting and comparison

Instead of using an a priori Bayesian model (see S1 Text #3 “Bayesian model”) to generate regressors of reward/effort predictions for the behavioral and neural regression analysis, it is also possible to derive these from reinforcement-learning models that are fitted to participants’ choice data (similar to analyses that we described previously [27,79]). We also fitted this class of models to ensure that our Bayesian model was appropriate, i.e., that it at least provided an equally good fit to the data. In short, the model consisted of three main components: firstly, the model had predictions of the mean reward/effort magnitudes underlying both outcomes. These were updated on every trial using a reinforcement-learning algorithm:

$$Prediction_{t}=Prediction_{t-1}+\alpha *PE_{t-1}$$

with

$$PE_{t-1}=Outcome_{t-1}-Prediction_{t-1}$$

where α was the learning rate. Thus, the learning rate was a measure of how much participants updated their reward/effort magnitude prediction when the reward/effort magnitude outcome differed from their expectation (i.e., depending on the PE). We fitted models that differed in their number of learning rates: they either shared the same learning rate for reward and effort, or they had separate learning rates. Finally, we also used a model with no fitted learning rate that instead used the predictions for reward and effort derived from the Bayesian model.

Secondly, the model then combined these reward/effort magnitude predictions together with the reward probabilities (explicitly shown to participants on each trial on the screen) to calculate how valuable each option was (i.e., their utility). Similar to [79], we considered different ways in which utility could be computed: optimally, probability and reward should be integrated multiplicatively:

$$Utility_{OptionA}=\frac{1}{k}*Probability_{Reward}*MagPrediction_{Reward}-\frac{\lambda }{k}*MagPrediction_{Effort}$$

in which k was a normalization constant with k = 1+λ, and λ was the effort magnitude decision weight. Alternatively, participants might use a heuristic strategy in which they instead integrate probability and reward linearly (we previously found this to provide a better fit to similar data [79]):

$$Utility_{OptionA}=\frac{1}{k}*Probability_{Reward}+\frac{\gamma }{k}*MagPrediction_{Reward}-\frac{\lambda }{k}*MagPrediction_{Effort}$$

in which k was a normalization constant with k = 1+γ+λ, γ was the reward magnitude decision weight, and λ was the effort magnitude decision weight. The Utility for option B was computed in the same way.

Thirdly, the model then compared the utility of the two options to predict how likely participants would be to choose either, using a standard soft-max decision rule:

$$P\left(Option_{A}\right)=\frac{e^{\beta *Utility_{A}}}{e^{\beta *Utility_{A}}+e^{\beta *Utility_{B}}}$$

in which β reflected participants’ ability to pick the option with the higher utility (i.e., the inverse temperature).

The data from each participant were fitted individually using Matlab’s fminsearch routine, which adjusted the free parameters to minimize the difference between the predicted choice and the actual choice. To compare how well each model fitted the data, we performed model comparison using the Bayesian Information Criterion (BIC) summed across all participants. As this model comparison revealed the Bayesian model to provide the best fit to the data, we used it to generate regressors for all regression analyses (fMRI and behavior). However, we note that all findings and conclusions from the regression analyses remain the same when regressors are instead derived from the best-fitting Rescorla—Wagner model. This is due to the very high correlations between the regressors derived from the Bayesian and the best fitting Rescorla—Wagner model (r > 0.99).

### Reward learning interference

To assess whether citalopram protected reward learning from interference, we performed a logistic regression analysis assessing how well information to be learnt from one trial (in the form of PEs) was used in the next trial when there was interference or when there was no interference (analysis bGLM2, see S11 Fig for correlations between the regressors). The regression predicted participant’s decisions to “stay” (choose the same option again as on the last trial) or “switch” (choose the alternative option compared to the last trial). Coding decision as “stay/switch” makes the behavioral analyses similar to the neural analyses: a regressor that is “chosen” in the outcome phase of one trial (fMRI) is a regressor favoring “stay” on the next trial (behavioral analysis). Equally an “unchosen” regressors favors “switch” on the next trial. As main regressors of interest to measure interference, we included interaction terms between the last trial’s relative (“stay” minus “switch”) RPEs and the two factors that could produce interference: the relative EPEs (positive values indicating higher, i.e., less favorable EPEs) and whether reward was real (coded as +1) or hypothetical (coded as −1). For this, we z-score normalized each regressor separately and then multiplied each interfering factor with the relative RPEs. As regressors of no interest, we included the explicitly shown (at the time of choice) relative probabilities, the relative reward and effort magnitude predictions, the relative reward and effort PEs, and the reward type (real versus hypothetical):

$$\begin{array}{l} Y_{bGLM2}=\beta_{0}\;+\beta_{1}RPE_{t-1}*RewardType_{t-1}+\beta_{2}RPE_{t-1}*EPE_{t-1}+\beta_{3}RewardProbability_{t}+\beta_{4}RewardMagnitudePrediction_{t-1}+\beta_{5}EffortMagnitudePrediction_{t-1}+\beta_{6}RewardType_{t-1}+\beta_{7}RPE_{t-1}+\beta_{8}EPE_{t-1} \end{array}$$

β_1_ and β_2_ were the two regression weights of interest, measuring the interference effects of reward type and effort learning on reward learning. If there is no interference by reward type, the regression weights should be (on average) zero, while if there is interference, β_1_ should be positive (if reward learning is worse when reward is only hypothetical). Similarly, for interference by EPE, if there is no interference, β_2_ should be (on average) zero. While if there is interference (i.e., if reward learning is worse when EPEs are high), β_2_ should be negative (as EPE increases the probability of switch rather than stay decisions). For Fig 5A, β_2_ has been sign reversed for illustration purposes so that for both β_1_ and β_2_, positive values indicate more interference. PEs and predictions were again obtained from the same Bayesian learning model as described for the analysis of the neural data; also, note that very similar results were found using regressors derived from a fitted learning model instead.

To further illustrate the results of analysis bGLM2 more intuitively, we compared to what extent participants could use RPEs on trials when reward was real or hypothetical using a regression analysis (bGLM3a). We included as our two regressors of interest relative RPEs (in favor of “stay” minus in favor of “switch”) separately on trials when reward was real or hypothetical. As regressors of no interest, we also included the relative reward probabilities (which were displayed to participants on each trial), the relative reward and effort predictions, the relative EPEs (also split into trials where reward was real or hypothetical), and reward type (real versus hypothetical):

$$\begin{array}{llll} Y_{bGLM3a}=\beta_{0}\;+\beta_{1}RPEwhenRealReward_{t-1}+\beta_{2}RPEwhenHypoth.Reward_{t-1} & +\beta_{3}EPEwhenRealReward_{t-1}+\beta_{4}EPEwhenHypoth.Reward_{t-1} & +\beta_{5}RewardProbability_{t}+\beta_{6}RewardMagnitudePrediction_{t-1} & +\beta_{7}EffortMagnitudePrediction_{t-1}+\beta_{8}RewardType_{t-1} \end{array}$$

To assess whether RPEs were used differently by the two groups when reward was real or hypothetical, we compared β_1_ and β_2_ using *t* tests. To assess whether EPEs were used differently when reward was real or hypothetical (S8 Fig), we compared β_3_ and β_4_ using *t* tests.

Similarly, we tested to what extent participants could use RPEs on trials when EPEs were particularly favorable or unfavorable (i.e., surprisingly high effort) in analysis bGLM3b. For this, we included as our two regressors of interest relative RPEs separately on the quartile of trials with most favorable and the quartile of trials with most unfavorable EPEs (the split into quartiles was necessary as the relative EPE regressor was continuous, rather than categorical). As regressors of no interest, we also included the relative RPEs in the remaining trials (i.e., the half of trials when relative EPE was neither high nor low), the relative reward probabilities, the relative reward and effort predictions, the relative EPE, and the reward type.

$$\begin{array}{lllll} Y_{BGLM3b} & =\beta_{0}\;+\beta_{1}RPEwhenhighEPEquartile_{t-1}+\beta_{2}RPEwhenlowEPEquartile_{t-1} & +\beta_{3}RPEwhenmidEPEhalf_{t-1}+\beta_{4}EPE_{t-1}+\beta_{5}RewardProbability_{t} & +\beta_{6}RewardMagnitudePrediction_{t-1}+\beta_{7}EffortMagnitudePrediction_{t-1} & +\beta_{8}RewardType_{t-1} \end{array}$$

We compared the resulting regression weights of the two groups for RPEs when EPEs were particularly high or low (β_1_ and β_2_) using *t* tests.

## Supporting information

S1 Fig RPE group differences are robust to modeling choices. Regressors for reward prediction errors (RPEs) used in the main text were derived from a Bayesian optimal observer model. Alternatively, regressors could be generated from a fitted reinforcement-learning model. The best fitting model of this type (see Fig 2 in main text) produced regressors that were very similar to the ones from the Bayesian model (r>0.99). Here, we instead simulated regressors based on learning rates between 0.1 and 0.6 (for comparison, the fitted learning rates for model M1 were: placebo = 0.38, citalopram = 0.31, see S7 Fig). This allowed us to test whether our results could be explained away by a mismatch between the learning rates of the Bayesian learner and the true learning rates of the placebo group. A consequence of such a mismatch would be that while the placebo group only appears to have very weak or no RPEs with the regressors derived from the Bayesian model, it might show stronger RPEs with regressors generated using a different learning rate. We do not find this to be the case: A-D show plots of time courses of the regression coefficients of RPEs (on BOLD activity) at different simulated learning rates. At all learning rates from 0.1 to 0.6, we always observe the same pattern of the citalopram group (dark green) showing stronger RPEs than the placebo group (light green): ANOVA, omnibus main effect of group, A (alpha = 0.1): F(1,27) = 7.9, p = 0.009; B (alpha = 0.2): F(1,27) = 10.0, p = 0.004; C (alpha = 0.4): F(1,27) = 7.6, p = 0.01; D (alpha = 0.6): F(1,27) = 7.6, p = 0.01). Data for individual participants can be found in S7 Data.
(TIFF)

S2 Fig Effort prediction error signals in regions that activated with relative effort magnitude outcomes. A) We identified areas in the outcome phase that increased in activity with the relative (chosen minus unchosen option) effort magnitude outcomes (analysis fGLM1). We identified a total of six areas in this contrast (for table of coordinates see S2B Table). The relative effort magnitude outcome signal (from analysis fGLM2) did not differ between the two groups (ANOVA, testing for a group difference across all areas: F(1,27) = 0.29, p = 0.60). B) Shows the time course of the correlations in these ROIs between the effort prediction error (EPE) of the chosen option and the neural BOLD signal for the placebo (light red) and the citalopram (dark red) groups (analysis fGLM2). Across areas, EPE led to a significant decrease in BOLD activity (ANOVA, main effect of EPE on BOLD across all six areas, including all participants: F(1,27) = 17.70, p<0.001). While there was no group difference across all of these areas (ANOVA, group difference across all areas: F(1,27) = 1.43, p = 0.24), there was a stronger EPE in some areas (ANOVA, interaction area x group F(5,135) = 2.45, p = 0.037). Follow-up t-tests revealed that citalopram enhanced EPEs selectively in dACC (t(27) = 3.01, p = 0.006). The ventral striatum is also shown for illustration as this area has been of general interest in studies looking at learning. There were again no group differences (t(27) = -0.087, p = 0.93). All results in A) are cluster-corrected at p<0.05. Abbreviations: dorsal anterior cingulate cortex (dACC), anterior insula/ frontal operculum (aIns), anterior prefrontal cortex (aPFC), parietal cortex (Parietal, IPL_C [79]), ventral striatum (striatum). Data for individual participants can be found in S7 Data.
(TIFF)

S3 Fig Non-cluster corrected results for group differences. To illustrate the pattern of group differences for reward and effort prediction errors (Fig 3, panels B and D) across the whole brain, this figure shows non-cluster corrected group difference activation maps (voxel threshold: p<0.05). A) Areas (in red) in which the citalopram group had stronger (i.e. more positive, compare Fig 3, panel B) reward prediction errors (chosen option) than the placebo group. We note that in addition to the areas discussed in the main text, the cluster in posterior cingulate was also significant using whole-brain cluster-correction. B) Areas (in blue) in which the citalopram group had stronger (i.e. more negative, compare Fig 3, panel D) effort prediction errors than the placebo group. Cross hairs show location of vmPFC (A) and dACC (B) ROIs used in to extract data for the analyses in the main manuscript (Figs 3 and 4). Brain maps can be found in S2 Data.
(TIFF)

S4 Fig Correlations of regressors used in the fMRI analysis fGLM1. The values are the mean of the absolute correlation values (r-values) across all participants. No r-values exceeded 0.44. Abbreviations: predicted reward/effort magnitude (PredRewMag, PredEffMag), reward type on the last trial before the current decision phase (Last reward typet-1), reward/effort prediction error (RPE, EPE), reward/effort magnitude outcome (RewMagOutcome, EffMagOutcome), option that was chosen by the participant (C), option that was not chosen, or ‘unchosen’ (UC). Data for individual participants can be found in S7 Data.
(TIFF)

S5 Fig Citalopram does not affect generic BOLD responses. In a control analysis (like fGLM2, with an additional constant regressor for the outcome phase in each trial, which is shown here), we tested whether citalopram might have increased general BOLD responses during the outcome phase. This was not the case (ANOVA, main effect of group: F(1,27) = 1.07, p = 0.31; area x group interaction: F(5.19,140.01) = 0.63, p = 0.69). This suggests that the increase in RPE/EPE (Fig 3, main text) was indeed very specific and not secondary to an effect of citalopram on the vasculature. Data for individual participants can be found in S7 Data.
(TIFF)

S6 Fig Procedure for time course analyses. In the following, we will illustrate the procedure used for time course analyses by looking at the impact of the mathematical reward prediction error (RPE) on brain activity in the ventromedial prefrontal cortex (vmPFC). A) Behavioral regressors. A Bayesian model (see S1 Text #3) was applied to the reward outcome values of each option shown in (1). In this way, reward predictions for each trial were created and these were sorted by which option was ‘chosen’ or ‘unchosen’ (i.e. the alternative option). The RPE was then computed by subtracting the reward prediction (expected reward) from the reward outcome on each trial (3). B) Neural data. We extracted neural data (BOLD) from a spherical region of interested ((4), ROI in white, here placed in vmPFC) based on the peak of an orthogonal activation contrast (here: main effect of activating more with real compared to hypothetical reward). As can be seen in the figure, there were sometimes large activation clusters spanning several different brain regions. If this was the case, we selected an activation peak that was well within the brain area of interest. The data from all voxels in the ROI was extracted and averaged (after standard pre-processing, see S1 Text #5 ‘MRI’). For illustration, we show the (normalized) BOLD samples recorded in the first 600s (time along the y-axis) of the experiment (black circles) for one participant, together with indicators (blue triangles) when the outcome phases of the first 6 trials of the experiment occurred. The data was then up-sampled tenfold and cut into epochs starting at the beginning of the outcome phase (5). C) Correlations between RPE regressor and BOLD. Each time-point of this up-sampled time course was then subjected to a GLM including for example the RPE as main regressor of interest (6), in addition to other confound regressors (see for example fGLM2 in Methods of main text). Thus we obtained a regression weight for each time point indicating the impact of RPE on brain activity. This was repeated for each participant and the resulting data was averaged across participants ((7), data shown here is from the citalopram group only). To test whether for example the two groups differed in the impact of RPE on BOLD in vmPFC, we employed a leave-one-out procedure in order to find the best alignment of the hemodynamic response function (hrf, 8), as described previously [27]. Specifically, for each participant, we used the data from all but that participant of his/her group (placebo or citalopram) to determine the absolute peak (i.e. either the strongest peak or trough) of the time course in a window between 6s and 12s. We then aligned the peak of a canonical hrf (grey) to this peak (black). The hrf was made using gammapdf in matlab, with values α = 7^2^/3^2^, β = 7/3^2^. We then multiplied the aligned hrf with the omitted participant’s time course and summed up the resulting values to obtain one value per participant for each regressor of interest (here RPE). The reason for employing the leave-one-out procedure, rather than employing a fixed delay of the hrf of e.g. 6s, was that it has been shown previously that hrf delays can vary strongly between different brain regions [33], so that a generic delay of 6s might not capture activity well in some brain areas. A leave-one-out procedure can correct for this in a non-biased way. This step was then repeated for each participant (9). Finally, we compared values between the groups using t-tests or ANOVAs as appropriate.
(TIFF)

S7 Fig Additional measures of behavioral learning. A) To obtain behavioral measures of overall learning, i.e. independent of interference effects (Fig 5), we performed a regression analysis (bGLM4, see S1 Text #4 ‘Behavioral supplementary regression analyses’) predicting whether participants repeated the same choice as on the previous trial (‘stay’) or ‘switched’ to the alternative choice. Here, learning was captured in the form of prediction errors (compare Fig 5). Participants in both groups were influenced by the relative (in favor of the ‘stay’ minus in favor of the ‘switch’ choice, st-sw) reward and effort prediction errors (both p<0.001), which we used therefore as a measure of learning. However, the groups did not differ (RPE: t(27) = -1.15, p = 0.26; EPE: t(27) = 0.23, p = 0.82). Additionally, and similarly to the neural results (Fig 3), the groups did not differ in their reward or effort sensitivity, i.e. in how much past rewards or effort influenced their choices (Reward prediction_t-1_: t(27) = -1.23, p = 0.23; Effort prediction_t-1_: t(27) = 1.14, p = 0.27). B) Next, we texted whether this measure of learning correlated with neural learning related activity (S1 Text #6 ‘Correlations between neural and behavioral prediction errors’). Across all participants in the two groups, the behavioral regression weight of the relative EPE correlated with the neural regression weight of relative EPE in several areas, including dorsal anterior cingulate cortex, dACC (Bi, Z = 3.93, MNI x = -2, y = 30, z = 38) and dorsolateral prefrontal cortex (Z = 3.46, x = 30, y = 40, z = 30, extending to anterior prefrontal cortex); results are whole-brain cluster-corrected (p<0.05). For illustration, Bii) shows a scatter plot of the same results from dACC. These results suggests that between-participant variations in the representation of effort learning signals (at the time of learning) in various brain areas relate to differences in how much participants use these learning signals when making decisions. We did however not find the same correlations between relative behavioral and neural RPEs on a whole-brain level. This is potentially because, as discussed at length in the main text, reward learning was subject to various kinds of interference when reward outcomes were hypothetical as opposed to real and when reward outcomes were accompanied by high EPEs. These important differences between different outcomes were not examined in this analysis although they are the focus of analyses in the main text. An alternative way of measuring learning is shown in C): Instead of including reward or effort PEs in a logistic regression to estimate learning, it is also possible to fit computational learning models (model ‘Rew/Eff LRs—Add’, see Fig 2C). From these models, learning is measured in the form of learning rates. We found that the learning rates estimated from the Rescorla-Wagner model correlated strongly with the regression weights for reward and effort PEs (from bGLM4 above): Ci) The regression weights for relative RPEs correlated with the estimated reward learning rates (r = 0.35, p = 0.007, nonparametric test). This effect remained significant after controlling for general sensitivity to reward, estimated by the regression weight for relative reward magnitude prediction (r = 0.43, p = 0.021). In contrast, the regression weights for relative RPEs did not correlate with the learning rate for effort (r = 0.19, p = 0.16, nonparametric test). Cii) The regression weights for relative EPEs correlated with the estimated effort learning rates (r = -0.47, p<0.001, nonparametric test). This effect remained significant after controlling for general sensitivity to effort, estimated by the regression weight for relative effort magnitude (r = -0.64, p<0.001). Again, the regression weights for relative EPEs did not correlate with the learning rate for reward (r = -0.01, p = 0.96, nonparametric test). This shows that our measures of learning in the form of regression weights for reward and effort PEs are strongly related to measures of learning in the form of the more commonly used learning rates. The parameters obtained from the Rescorla-Wagner model for the two groups were: inverse temperature: placebo: 0.04, citalopram: 0.05, p = 0.22; relative reward magnitude decision weight: placebo: 0.37, citalopram: 0.42, p = 0.51; relative effort magnitude decision weight: placebo:0.35, citalopram:0.27, p = 0.28; learning rate for reward: placebo = 0.38, citalopram = 0.31, p = 0.50; learning rate for effort: placebo = 0.47, citalopram = 0.41, p = 0.58. Data for individual participants can be found in S7 Data.
(TIFF)

S8 Fig Interference on behavioral effort learning. In analyses bGLM3a+bGLM5, we assessed whether the extent to which effort prediction errors (EPEs) affected participants decisions to ‘stay’ (i.e. chose the option again as on the previous trial) or ‘switch’ to the alternative option varied as a function of potential interfering factors. Shown are the regression weights for how much relative (in favor of the ‘stay’ minus in favor of the ‘switch’ option) EPEs on one trial impacted decisions to stay or switch on the next trial. Negative regression weights mean that when EPEs are high for the option that has been chosen compared to the alternative, participants are more likely to switch to the alternative on the next trial. We found that, in contrast to RPEs (Fig 5), EPEs were not affected by either interference from reward type or RPEs: A) The two groups could use EPEs equally well when rewards were real (group difference: t(27) = -0.03, p = 0.98) or hypothetical (group difference: t(27) = 0.41, p = 0.69). B) When EPEs were examined separately for when RPEs were favorable (i.e. the quartile of trials with most positive relative RPEs), the groups could use EPEs equally well (group difference: t(27) = -1.7, p = 0.10). Similarly, when RPEs were unfavorable (i.e. the quartile of trials with the most negative relative RPEs), both groups could use EPEs and did not differ in their use of EPEs (group difference: t(27) = 0.44, p = 0.66). Data for individual participants can be found in S7 Data.
(TIFF)

S9 Fig Influence of citalopram on RPEs is general. We tested whether effects of citalopram on neural learning signals were always present or only found in situations of interferences. For this, we added to the regression analysis fGLM2, analogous to behavioral regression bGLM2 (Fig 5), interaction terms between RPE a reward type (B) and between RPE and EPE (C). We found that, as before (Fig 3B), the citalopram group had a larger RPE signal (A, F(1,27) = 7.8, p = 0.009). However, we did not find neural learning signals (RPEs) to be affected by interfering factors, i.e. reward being only hypothetical (B, F(1,27) = 0.55, p = 0.47) or EPEs being particularly salient (C, F(1,27) = 0.35, p = 0.56). Data for individual participants can be found in S7 Data.
(TIFF)

S10 Fig Effort exertion. As behavioral measure in the effort phase, we collected participants’ rates of clicking with the trackball mouse on each trial. Effort exertion behavior did not differ between the groups. First, they did not differ in their average clicking rates (t(27) = -0.01, p = 1.00). Second, we tested whether the different reward factors influenced the clicking rate using a regression analysis (eGLM1). We found that the reward of the option that was chosen (one-sample t-test on combined data from both groups: t(28) = 3.23, p = 0.003) and the irrelevant reward information (i.e. the average of the regression weights for the reward of the option that was not chosen and the reward type, real vs. hypothetical, one-sample t-test on combined data from both groups: t(28) = 2.46, p = 0.02) increased the clicking rate across both groups. However, this did not differ between the groups (both p>0.5). Furthermore, both groups also almost always completed the effort phase: the placebo group failed to complete the effort phase on 0.6±0.3% of trials inside and on 0.2±0.1% of trials outside the scanner, while the citalopram group failed to complete the effort phase on 0.7±0.3% of trials inside and on 0% of trials outside the scanner. Data for individual participants can be found in S7 Data.
(TIFF)

S11 Fig Correlations of regressors used in the behavioral regression analysis (analysis bGLM1). The values are the mean of the absolute correlation values (r-values) across all participants. No r-values exceeded 0.38. Abbreviations: In favor of the ‘stay’ option (St, i.e. in favor of repeating the last trial’s choice), in favor of the ‘switch’ option (Sw, i.e. in favor of selecting the alternative option compared to the last trial), predicted effort/reward magnitude on trial t-1 (PredEffMag_t-1_, PredRewMag_t-1_), effort/reward prediction error on trial t-1 (RPE_t-1_, EPE_t-1_), interaction between relative reward prediction error and effort prediction error on trial t-1 (RPE_t-1_(St-Sw) x EPE_t-1_(St-Sw)), interaction between reward prediction error and reward type on trial t-1 (RPE_t-1_(St-Sw) x Reward type_t-1_). Data for individual participants can be found in S7 Data.
(TIFF)

S1 Table Participant demographics and questionnaire scores. Tables show baseline measurements (A), measurements at the time of the test, i.e. after two weeks of citalopram or placebo (B) and difference scores between the time of test and baseline (C). The number of participants for who measures were available is given for each measurement (# Subjects), together with the mean value and standard error of the mean of each group and the resulting p-values of two-tailed t-tests comparing the two groups. Abbreviations and questionnaires used: Beck’s Depression Inventory (BDI, Beck et al. [80]), State-trait anxiety inventory [81], Positive and negative affect schedule (Panas, Watson et al. [82]), Cloninger scale [83], Bond-Lader scale (BL, Bond and Lader [84]. Blinding of participants to the drug condition was assessed by asking them informally which group they believed to be in (positive values indicate citalopram, negative values placebo) and how certain they were (10 = very certain, 0 = very uncertain). P-values are the results of two-sample two-tailed t-tests, not corrected for multiple comparisons. While we note that the groups differed in the contentedness measure of the BL scale at the ‘test’ time point, this would not be significant after correction for multiple comparisons. Also note that inclusion of the questionnaire measures does not affect any of the results reported in the main paper. Data for individual participants can be found in S6 Data.
(XLSX)

S2 Table Brain activity in the outcome phase. A) Areas activating more to real than hypothetical reward (i.e. reward type). B) Areas activating with the relative effort magnitude outcome (chose minus unchosen option). All results were obtained from analysis fGLM1 and were significant with whole-brain cluster-correction p<0.05 (voxel inclusion threshold: z>2.3). Where relevant references for area labels are: (1):[79], (2):[85], (3):[86].
(XLSX)

S1 Text Supporting methods. (DOCX)

S1 Data Table with individual participants’ regression weights (Fig 2B) and model fits (Fig 2C). (XLSX)

S2 Data This folder contains all MRI contrast maps (Figs 3+4, S2 and S3 Figs), both thresholded (i.e., corrected for multiple comparison using cluster correction) and non-thresholded. The maps are in NIfTI format and can be opened with freely available data viewers such as FSLView or MRIcron.
(ZIP)

S3 Data Table with FMRI time courses for individual participants for reward prediction errors (Fig 3B) and effort prediction errors (Fig 3D). (XLSX)

S4 Data Table with FMRI time courses for individual participants for reward magnitude outcomes (Fig 4A) and effort magnitude outcomes (Fig 4B). (XLSX)

S5 Data Table with individual participants’ regression coefficients (Fig 5A–5C). (XLSX)

S6 Data Table with individual participants’ questionnaire scores, measured before administration of drug/placebo and after two weeks of drug/placebo (S1 Table). (XLSX)

S7 Data Tables for all supplementary figures. (XLSX)

## Abbreviations

BIC: Bayesian Information Criterion
dACC: dorsal anterior cingulate cortex
EPE: effort prediction error
fMRI: functional magnetic resonance imaging
OCD: obsessive-compulsive disorder
PE: prediction error
ROI: regions of interest
RPE: reward prediction error
SSRI: selective serotonin reuptake inhibitor
vmPFC: ventromedial prefrontal cortex
