# Supplementary figures and images for: Beyond negative valence: 2-week administration of a serotonergic antidepressant enhances both reward and effort learning signals
Source: PLoS Biol. 2017 Feb 16;15(2):e2000756. doi: 10.1371/journal.pbio.2000756 (PMC5331946; doi:10.1371/journal.pbio.2000756)

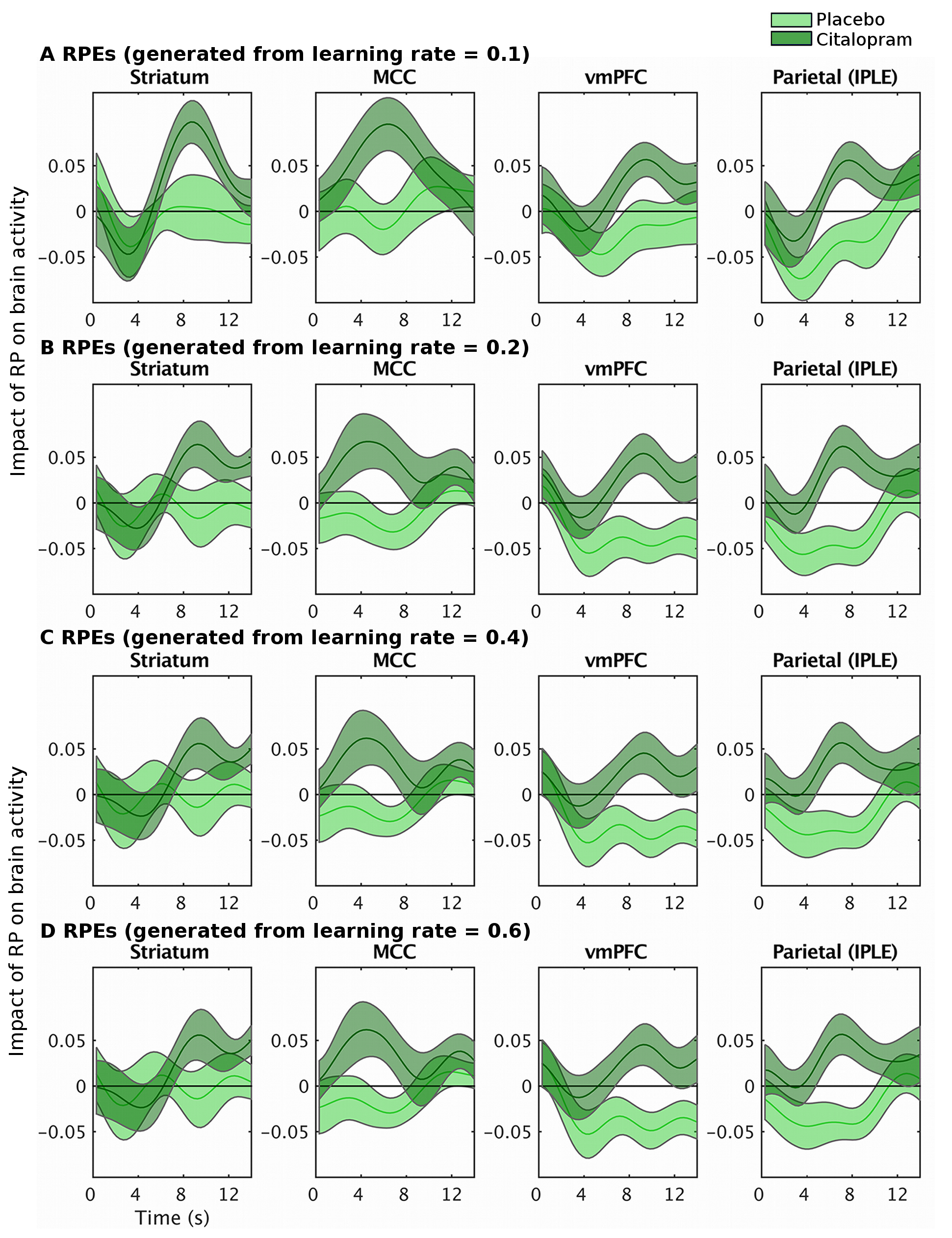

Supplement: S1 Fig — Regressors for reward prediction errors (RPEs) used in the main text were derived from a Bayesian optimal observer model. Alternatively, regressors could be generated from a fitted reinforcement-learning model. The best fitting model of this type (see Fig 2 in main text) produced regressors that were very similar to the ones from the Bayesian model (r>0.99). Here, we instead simulated regressors based on learning rates between 0.1 and 0.6 (for comparison, the fitted learning rates for model M1 were: placebo = 0.38, citalopram = 0.31, see S7 Fig). This allowed us to test whether our results could be explained away by a mismatch between the learning rates of the Bayesian learner and the true learning rates of the placebo group. A consequence of such a mismatch would be that while the placebo group only appears to have very weak or no RPEs with the regressors derived from the Bayesian model, it might show stronger RPEs with regressors generated using a different learning rate. We do not find this to be the case: A-D show plots of time courses of the regression coefficients of RPEs (on BOLD activity) at different simulated learning rates. At all learning rates from 0.1 to 0.6, we always observe the same pattern of the citalopram group (dark green) showing stronger RPEs than the placebo group (light green): ANOVA, omnibus main effect of group, A (alpha = 0.1): F(1,27) = 7.9, p = 0.009; B (alpha = 0.2): F(1,27) = 10.0, p = 0.004; C (alpha = 0.4): F(1,27) = 7.6, p = 0.01; D (alpha = 0.6): F(1,27) = 7.6, p = 0.01). Data for individual participants can be found in S7 Data. (TIFF) [file pbio.2000756.s001.tiff]

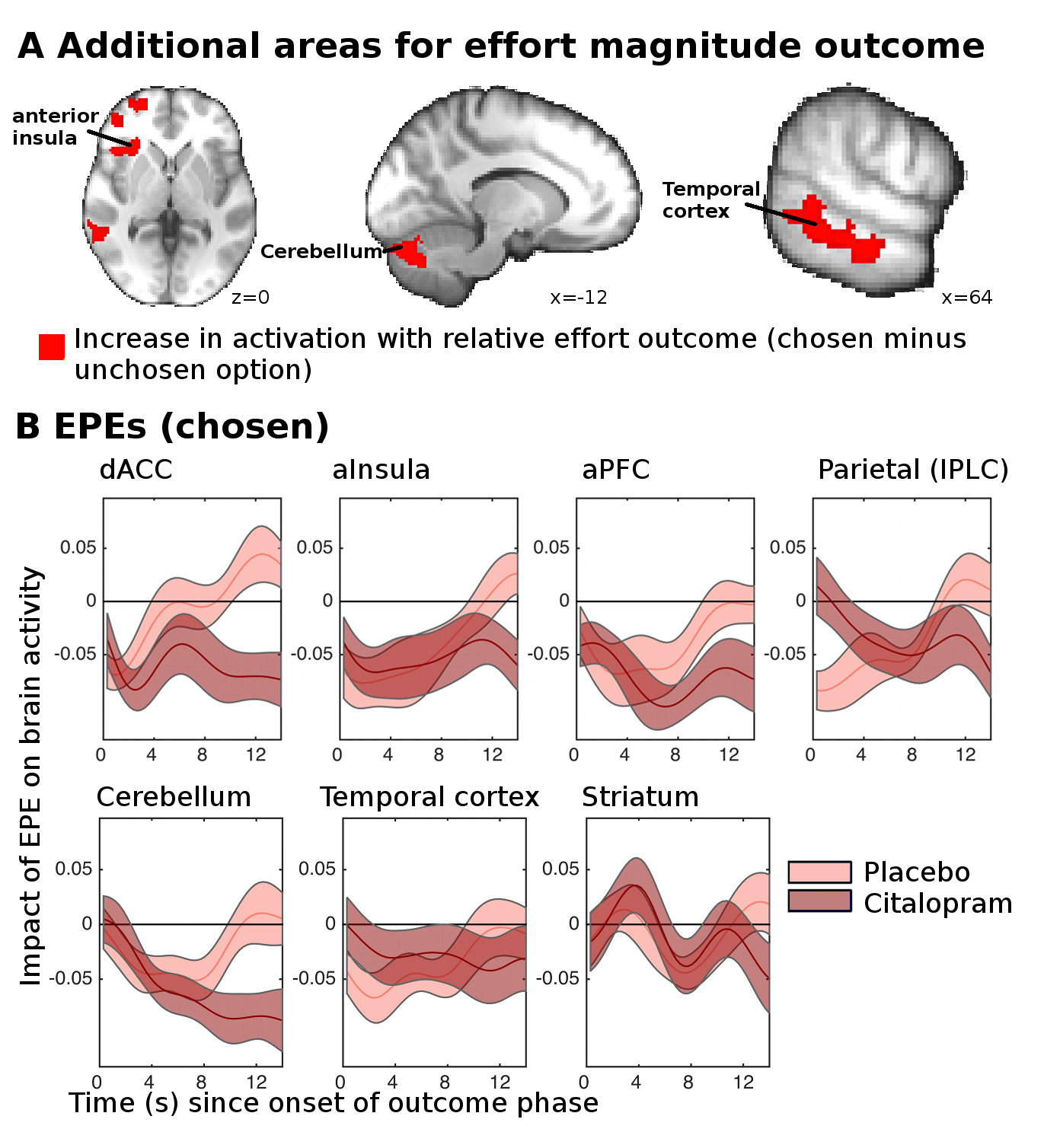

Supplement: S2 Fig — A) We identified areas in the outcome phase that increased in activity with the relative (chosen minus unchosen option) effort magnitude outcomes (analysis fGLM1). We identified a total of six areas in this contrast (for table of coordinates see S2B Table). The relative effort magnitude outcome signal (from analysis fGLM2) did not differ between the two groups (ANOVA, testing for a group difference across all areas: F(1,27) = 0.29, p = 0.60). B) Shows the time course of the correlations in these ROIs between the effort prediction error (EPE) of the chosen option and the neural BOLD signal for the placebo (light red) and the citalopram (dark red) groups (analysis fGLM2). Across areas, EPE led to a significant decrease in BOLD activity (ANOVA, main effect of EPE on BOLD across all six areas, including all participants: F(1,27) = 17.70, p<0.001). While there was no group difference across all of these areas (ANOVA, group difference across all areas: F(1,27) = 1.43, p = 0.24), there was a stronger EPE in some areas (ANOVA, interaction area x group F(5,135) = 2.45, p = 0.037). Follow-up t-tests revealed that citalopram enhanced EPEs selectively in dACC (t(27) = 3.01, p = 0.006). The ventral striatum is also shown for illustration as this area has been of general interest in studies looking at learning. There were again no group differences (t(27) = -0.087, p = 0.93). All results in A) are cluster-corrected at p<0.05. Abbreviations: dorsal anterior cingulate cortex (dACC), anterior insula/ frontal operculum (aIns), anterior prefrontal cortex (aPFC), parietal cortex (Parietal, IPL_C [79]), ventral striatum (striatum). Data for individual participants can be found in S7 Data. (TIFF) [file pbio.2000756.s002.tiff]

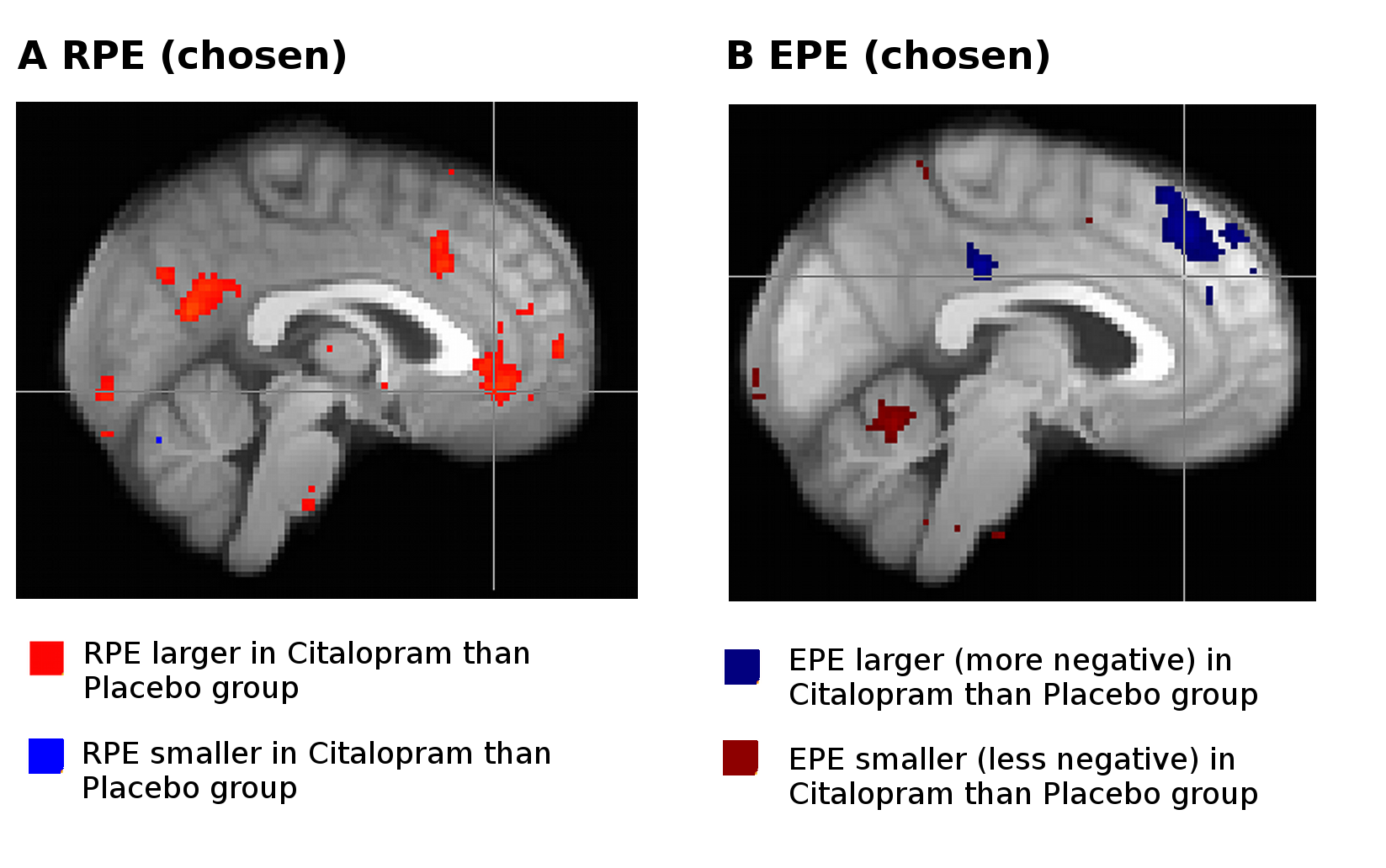

Supplement: S3 Fig — To illustrate the pattern of group differences for reward and effort prediction errors (Fig 3, panels B and D) across the whole brain, this figure shows non-cluster corrected group difference activation maps (voxel threshold: p<0.05). A) Areas (in red) in which the citalopram group had stronger (i.e. more positive, compare Fig 3, panel B) reward prediction errors (chosen option) than the placebo group. We note that in addition to the areas discussed in the main text, the cluster in posterior cingulate was also significant using whole-brain cluster-correction. B) Areas (in blue) in which the citalopram group had stronger (i.e. more negative, compare Fig 3, panel D) effort prediction errors than the placebo group. Cross hairs show location of vmPFC (A) and dACC (B) ROIs used in to extract data for the analyses in the main manuscript (Figs 3 and 4). Brain maps can be found in S2 Data. (TIFF) [file pbio.2000756.s003.tiff]

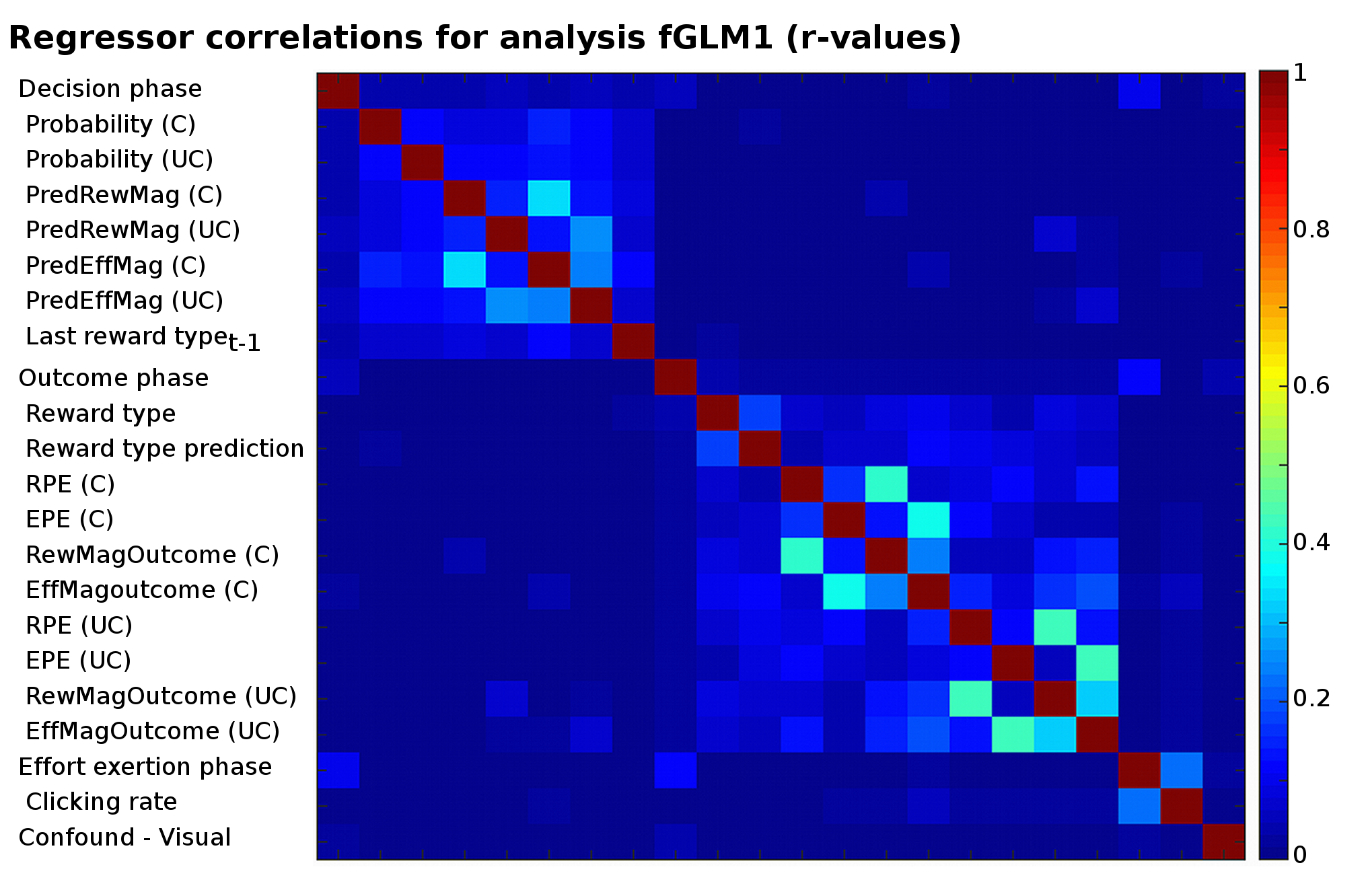

Supplement: S4 Fig — The values are the mean of the absolute correlation values (r-values) across all participants. No r-values exceeded 0.44. Abbreviations: predicted reward/effort magnitude (PredRewMag, PredEffMag), reward type on the last trial before the current decision phase (Last reward typet-1), reward/effort prediction error (RPE, EPE), reward/effort magnitude outcome (RewMagOutcome, EffMagOutcome), option that was chosen by the participant (C), option that was not chosen, or ‘unchosen’ (UC). Data for individual participants can be found in S7 Data. (TIFF) [file pbio.2000756.s004.tiff]

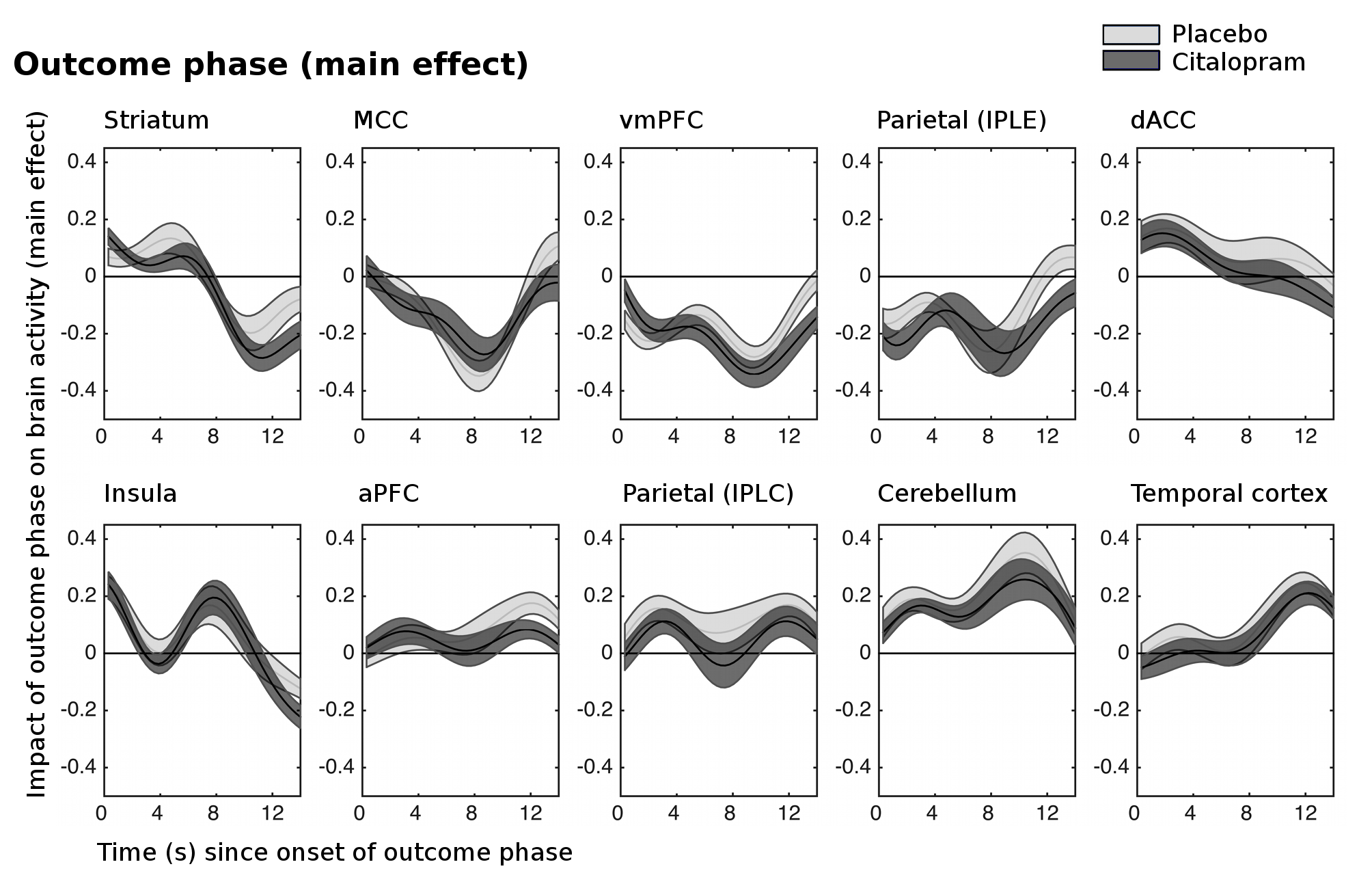

Supplement: S5 Fig — In a control analysis (like fGLM2, with an additional constant regressor for the outcome phase in each trial, which is shown here), we tested whether citalopram might have increased general BOLD responses during the outcome phase. This was not the case (ANOVA, main effect of group: F(1,27) = 1.07, p = 0.31; area x group interaction: F(5.19,140.01) = 0.63, p = 0.69). This suggests that the increase in RPE/EPE (Fig 3, main text) was indeed very specific and not secondary to an effect of citalopram on the vasculature. Data for individual participants can be found in S7 Data. (TIFF) [file pbio.2000756.s005.tiff]

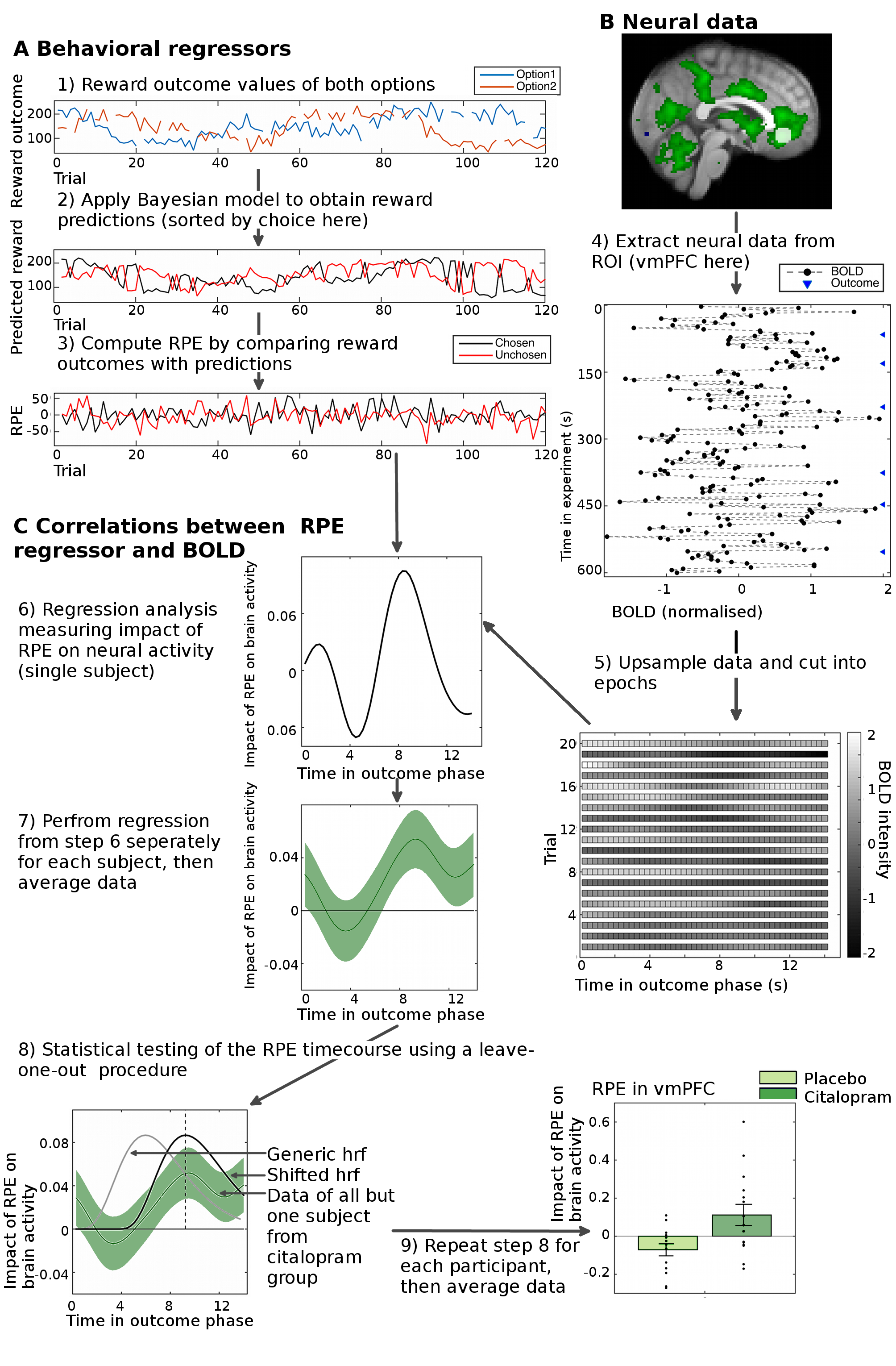

Supplement: S6 Fig — In the following, we will illustrate the procedure used for time course analyses by looking at the impact of the mathematical reward prediction error (RPE) on brain activity in the ventromedial prefrontal cortex (vmPFC). A) Behavioral regressors. A Bayesian model (see S1 Text #3) was applied to the reward outcome values of each option shown in (1). In this way, reward predictions for each trial were created and these were sorted by which option was ‘chosen’ or ‘unchosen’ (i.e. the alternative option). The RPE was then computed by subtracting the reward prediction (expected reward) from the reward outcome on each trial (3). B) Neural data. We extracted neural data (BOLD) from a spherical region of interested ((4), ROI in white, here placed in vmPFC) based on the peak of an orthogonal activation contrast (here: main effect of activating more with real compared to hypothetical reward). As can be seen in the figure, there were sometimes large activation clusters spanning several different brain regions. If this was the case, we selected an activation peak that was well within the brain area of interest. The data from all voxels in the ROI was extracted and averaged (after standard pre-processing, see S1 Text #5 ‘MRI’). For illustration, we show the (normalized) BOLD samples recorded in the first 600s (time along the y-axis) of the experiment (black circles) for one participant, together with indicators (blue triangles) when the outcome phases of the first 6 trials of the experiment occurred. The data was then up-sampled tenfold and cut into epochs starting at the beginning of the outcome phase (5). C) Correlations between RPE regressor and BOLD. Each time-point of this up-sampled time course was then subjected to a GLM including for example the RPE as main regressor of interest (6), in addition to other confound regressors (see for example fGLM2 in Methods of main text). Thus we obtained a regression weight for each time point indicating the impact of RPE on brain activ [file pbio.2000756.s006.tiff]

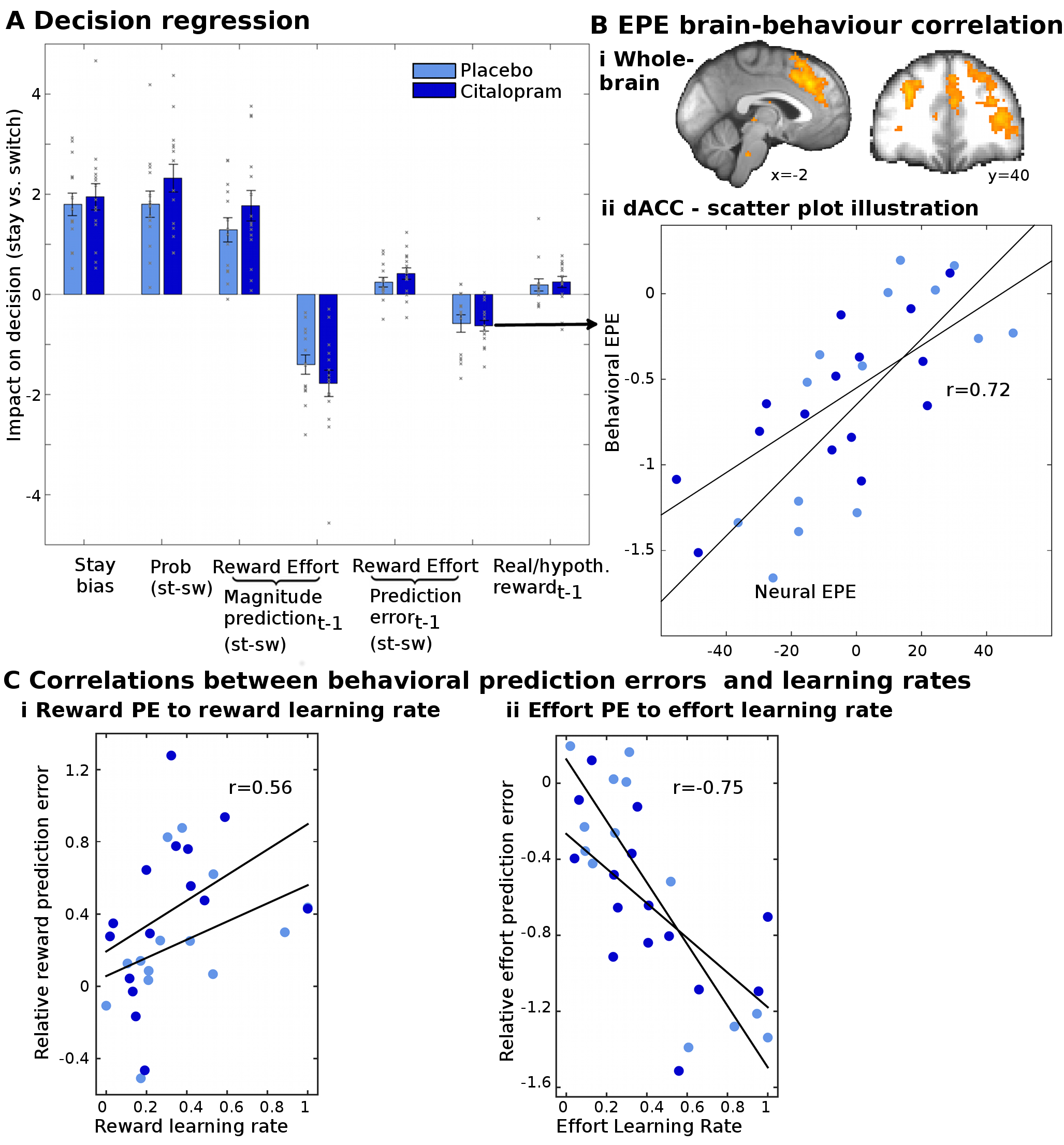

Supplement: S7 Fig — A) To obtain behavioral measures of overall learning, i.e. independent of interference effects (Fig 5), we performed a regression analysis (bGLM4, see S1 Text #4 ‘Behavioral supplementary regression analyses’) predicting whether participants repeated the same choice as on the previous trial (‘stay’) or ‘switched’ to the alternative choice. Here, learning was captured in the form of prediction errors (compare Fig 5). Participants in both groups were influenced by the relative (in favor of the ‘stay’ minus in favor of the ‘switch’ choice, st-sw) reward and effort prediction errors (both p<0.001), which we used therefore as a measure of learning. However, the groups did not differ (RPE: t(27) = -1.15, p = 0.26; EPE: t(27) = 0.23, p = 0.82). Additionally, and similarly to the neural results (Fig 3), the groups did not differ in their reward or effort sensitivity, i.e. in how much past rewards or effort influenced their choices (Reward predictiont-1: t(27) = -1.23, p = 0.23; Effort predictiont-1: t(27) = 1.14, p = 0.27). B) Next, we texted whether this measure of learning correlated with neural learning related activity (S1 Text #6 ‘Correlations between neural and behavioral prediction errors’). Across all participants in the two groups, the behavioral regression weight of the relative EPE correlated with the neural regression weight of relative EPE in several areas, including dorsal anterior cingulate cortex, dACC (Bi, Z = 3.93, MNI x = -2, y = 30, z = 38) and dorsolateral prefrontal cortex (Z = 3.46, x = 30, y = 40, z = 30, extending to anterior prefrontal cortex); results are whole-brain cluster-corrected (p<0.05). For illustration, Bii) shows a scatter plot of the same results from dACC. These results suggests that between-participant variations in the representation of effort learning signals (at the time of learning) in various brain areas relate to differences in how much participants use these learning signals when making decisions. We did however not find the sa [file pbio.2000756.s007.tiff]

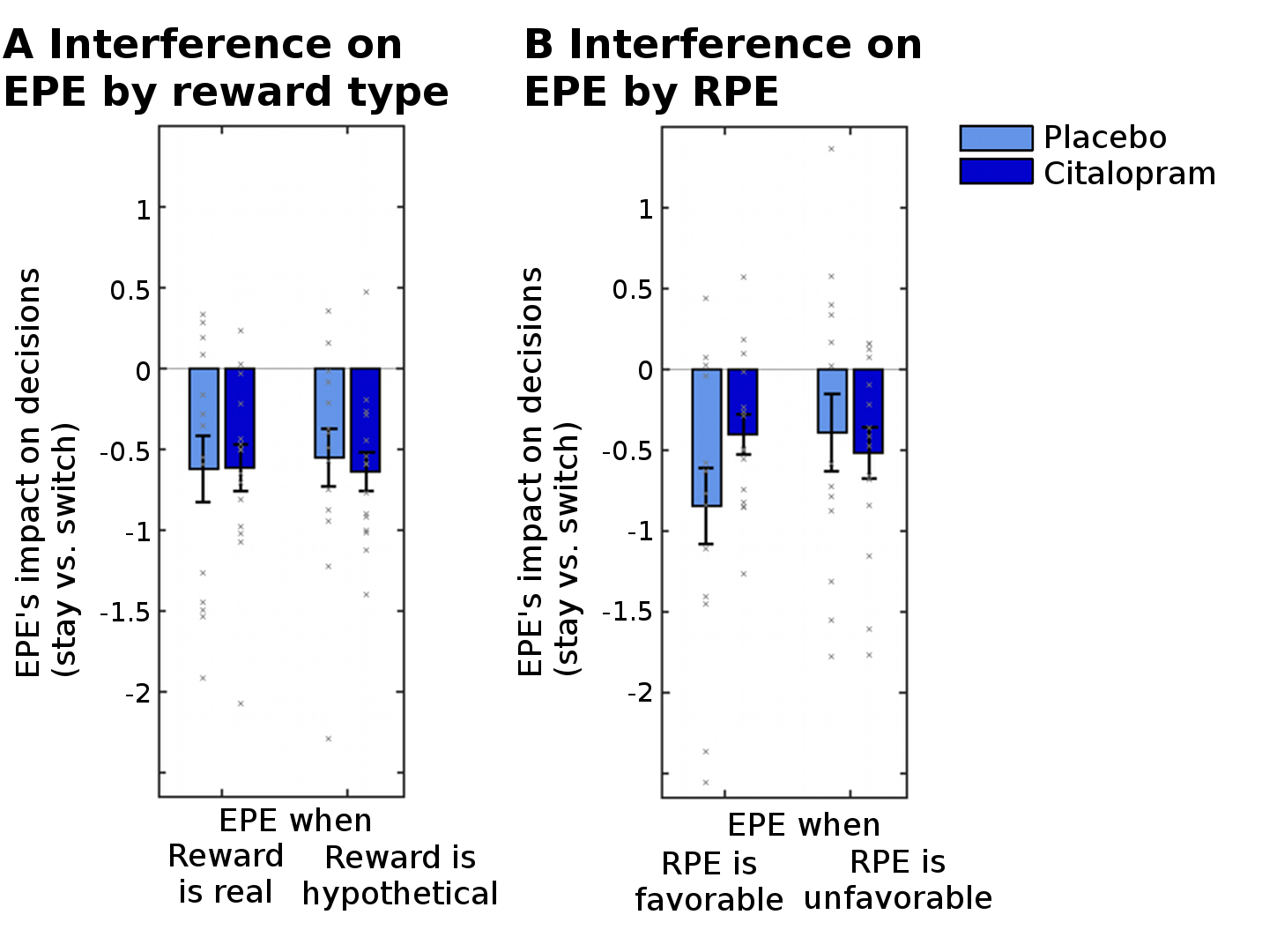

Supplement: S8 Fig — In analyses bGLM3a+bGLM5, we assessed whether the extent to which effort prediction errors (EPEs) affected participants decisions to ‘stay’ (i.e. chose the option again as on the previous trial) or ‘switch’ to the alternative option varied as a function of potential interfering factors. Shown are the regression weights for how much relative (in favor of the ‘stay’ minus in favor of the ‘switch’ option) EPEs on one trial impacted decisions to stay or switch on the next trial. Negative regression weights mean that when EPEs are high for the option that has been chosen compared to the alternative, participants are more likely to switch to the alternative on the next trial. We found that, in contrast to RPEs (Fig 5), EPEs were not affected by either interference from reward type or RPEs: A) The two groups could use EPEs equally well when rewards were real (group difference: t(27) = -0.03, p = 0.98) or hypothetical (group difference: t(27) = 0.41, p = 0.69). B) When EPEs were examined separately for when RPEs were favorable (i.e. the quartile of trials with most positive relative RPEs), the groups could use EPEs equally well (group difference: t(27) = -1.7, p = 0.10). Similarly, when RPEs were unfavorable (i.e. the quartile of trials with the most negative relative RPEs), both groups could use EPEs and did not differ in their use of EPEs (group difference: t(27) = 0.44, p = 0.66). Data for individual participants can be found in S7 Data. (TIFF) [file pbio.2000756.s008.tiff]

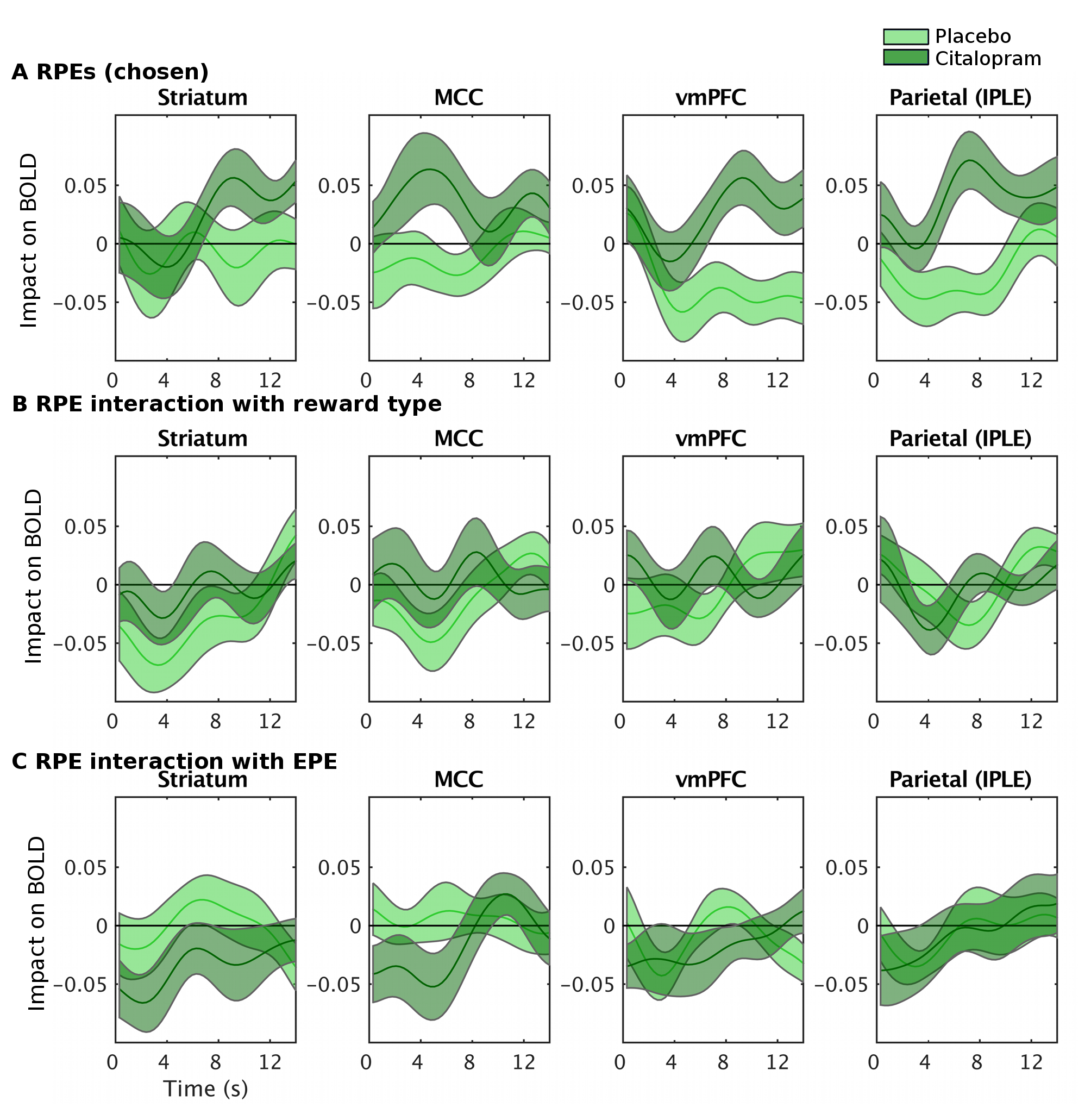

Supplement: S9 Fig — We tested whether effects of citalopram on neural learning signals were always present or only found in situations of interferences. For this, we added to the regression analysis fGLM2, analogous to behavioral regression bGLM2 (Fig 5), interaction terms between RPE a reward type (B) and between RPE and EPE (C). We found that, as before (Fig 3B), the citalopram group had a larger RPE signal (A, F(1,27) = 7.8, p = 0.009). However, we did not find neural learning signals (RPEs) to be affected by interfering factors, i.e. reward being only hypothetical (B, F(1,27) = 0.55, p = 0.47) or EPEs being particularly salient (C, F(1,27) = 0.35, p = 0.56). Data for individual participants can be found in S7 Data. (TIFF) [file pbio.2000756.s009.tiff]

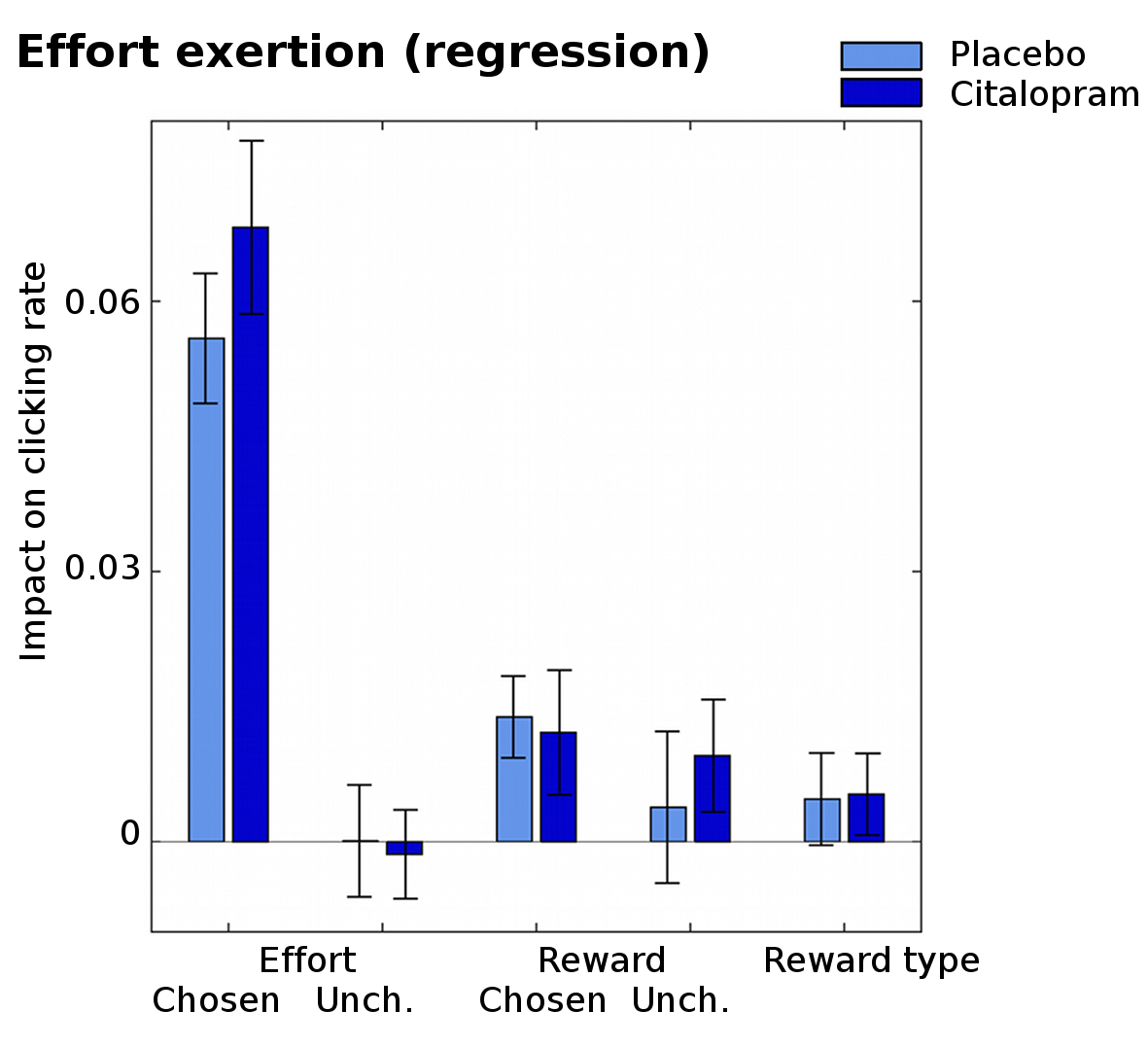

Supplement: S10 Fig — As behavioral measure in the effort phase, we collected participants’ rates of clicking with the trackball mouse on each trial. Effort exertion behavior did not differ between the groups. First, they did not differ in their average clicking rates (t(27) = -0.01, p = 1.00). Second, we tested whether the different reward factors influenced the clicking rate using a regression analysis (eGLM1). We found that the reward of the option that was chosen (one-sample t-test on combined data from both groups: t(28) = 3.23, p = 0.003) and the irrelevant reward information (i.e. the average of the regression weights for the reward of the option that was not chosen and the reward type, real vs. hypothetical, one-sample t-test on combined data from both groups: t(28) = 2.46, p = 0.02) increased the clicking rate across both groups. However, this did not differ between the groups (both p>0.5). Furthermore, both groups also almost always completed the effort phase: the placebo group failed to complete the effort phase on 0.6±0.3% of trials inside and on 0.2±0.1% of trials outside the scanner, while the citalopram group failed to complete the effort phase on 0.7±0.3% of trials inside and on 0% of trials outside the scanner. Data for individual participants can be found in S7 Data. (TIFF) [file pbio.2000756.s010.tiff]

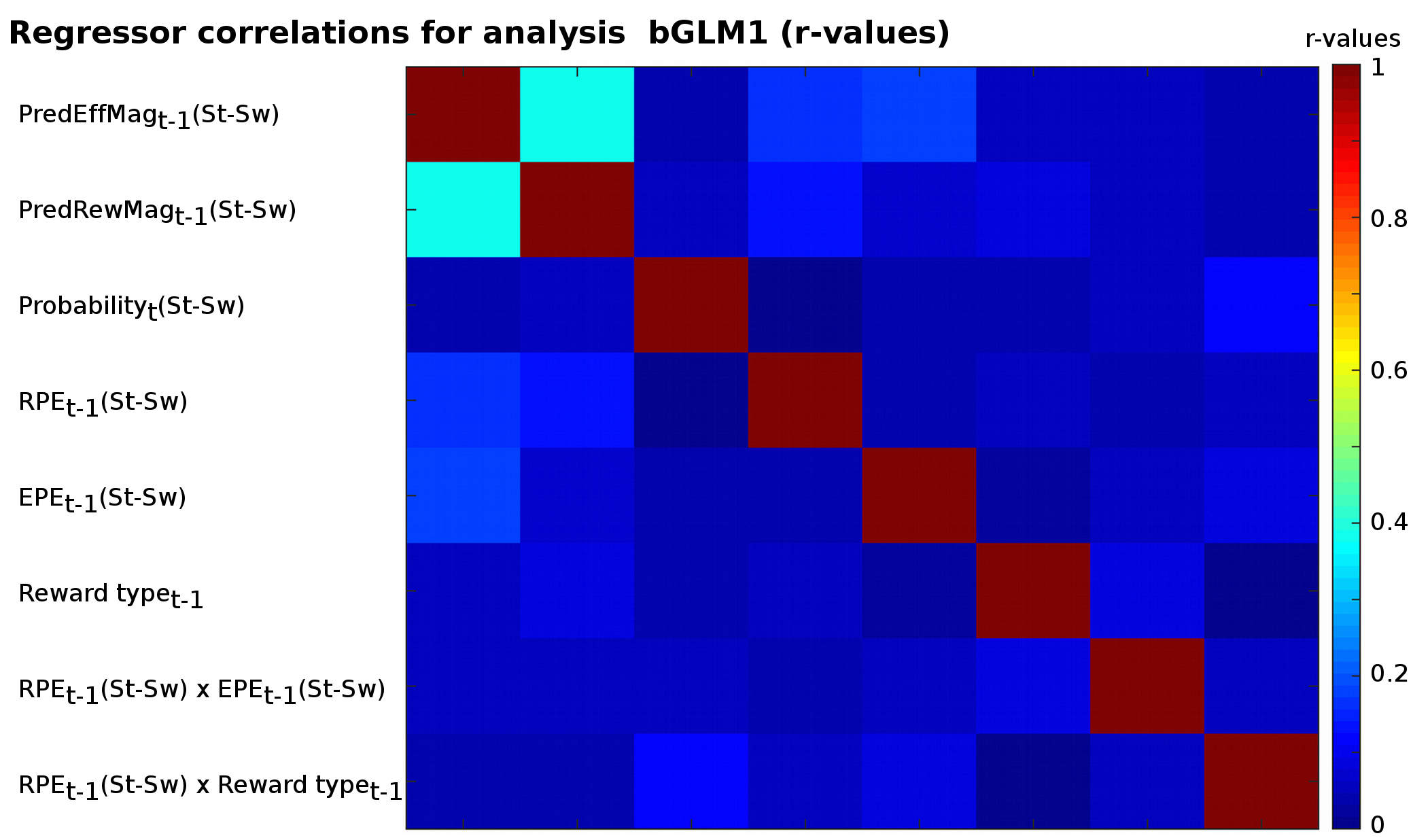

Supplement: S11 Fig — The values are the mean of the absolute correlation values (r-values) across all participants. No r-values exceeded 0.38. Abbreviations: In favor of the ‘stay’ option (St, i.e. in favor of repeating the last trial’s choice), in favor of the ‘switch’ option (Sw, i.e. in favor of selecting the alternative option compared to the last trial), predicted effort/reward magnitude on trial t-1 (PredEffMagt-1, PredRewMagt-1), effort/reward prediction error on trial t-1 (RPEt-1, EPEt-1), interaction between relative reward prediction error and effort prediction error on trial t-1 (RPEt-1(St-Sw) x EPEt-1(St-Sw)), interaction between reward prediction error and reward type on trial t-1 (RPEt-1(St-Sw) x Reward typet-1). Data for individual participants can be found in S7 Data. (TIFF) [file pbio.2000756.s011.tiff]
