# Supplementary material for: Beyond negative valence: 2-week administration of a serotonergic antidepressant enhances both reward and effort learning signals
Source: PLoS Biol. 2017 Feb 16;15(2):e2000756. doi: 10.1371/journal.pbio.2000756 (PMC5331946; doi:10.1371/journal.pbio.2000756)
Supplement: S1 Text — (DOCX) [file pbio.2000756.s014.docx]

**Supporting methods**

**1) Participants**

The study was approved by the local ethics committee. 46 healthy volunteers were recruited for the study screening session after providing written informed consent. They underwent psychiatric and medical screening before being included in the behavioral and MRI part of the study: They were included if they were right handed, they passed basic medical screening for current or past physical illness relevant to the study, were non- or light-smokers (<5 cigarettes a day), had taken no CNS-active medication during the last 8 weeks, were screened to be free of a past or present Axis 1 disorder on the Structured Clinical Interview for DSM-IV [1], had no first-degree relative with bipolar disorder, were fluent in English and had no counter-indications to FMRI. Female participants were neither pregnant nor breast-feeding. After the screening, 8 participants were excluded because of counter-indications to citalopram or the MRI scan, 6 participants were excluded because they were not sensitive to the effort (i.e. they did not find the effort task effortful or were willing to forfeit money to avoid the effort) and 2 participants dropped out before the MRI scan due to side-effects of citalopram. Of the participants taking part in the MRI session, data from one participant was excluded from analysis because he/she fell asleep during the MRI scan. There remained 15 participants in the citalopram group and 14 in the placebo group. Based on effect sizes reported in previous studies looking at the effects of prolonged SSRI administration on neural measures of learning in rodents [3] or on behavioral measures of learning in human patients with obsessive-compulsive disorder [4], our study had 84% power to detect an effect at alpha = 0.05. Participants were given identically looking beige capsules (obtained from Goerlich Pharma International GmbG) that either contained citalopram or placebo tablets (manufacturer: Rayonex Biomedical GmbH) in a double-blind between-subject design. Data was only unblinded after data collection finished except for the participants who dropped out because of side effects.

**2) Task description**

This task description has been adapted from [2]. We designed a learning task to measure whether citalopram affected learning about rewards or efforts. In the task, participants made repeated choices between two options with the aim of maximizing their monetary pay-off and minimizing the effort they needed to exert in an interleaved ‘effort phase’ (Fig. 1). On each trial there were three phases: first participants chose between two options (‘choice phase’), then they were shown the outcome of their choice (‘outcome phase’), then they had to exert the effort associated with the option they had chosen (‘effort phase’).

In the decision phase (Figure 1A), participants chose between two options using buttons on a trackball mouse. Each option had three independent attributes: a reward magnitude (reward points, later translated into monetary pay-off), an effort magnitude (amount of effort required in the effort phase), and a probability of receiving a real (rather than a hypothetical reward, see below). The probability of each option was randomly drawn (between 25% and 75%) and shown explicitly on the screen at the time of choice. In contrast, the reward and effort magnitudes of the options were not explicitly instructed and instead participants had to learn and track these slowly changing features of the two choices across trials. These magnitudes were drawn from normal distributions of which the means fluctuated pseudorandomly, slowly and independently over the course of the experiment between three levels (low, mid, high), Figure 1D. Participants were instructed to learn and keep track of the changing mean value of each magnitude across the experiment. Thus, the reward magnitudes the choices yielded constituted the only relevant reward information that could be tracked and learned, whereas the actual reward receipt and the probability thereof (whether the reward was real or hypothetical), should not have had any bearing on future decisions. Only one of the reward or effort magnitude means was drifting at any one time and each of the four magnitudes was at each mean level equally often.

We also note that including randomly drawn reward probabilities had the advantage of more clearly dissociating learning from decisions: if the task only had had reward and effort magnitude information, participants would have been able to make a decision for the next trial already at the time when the outcome information was revealed. In contrast, because they were still lacking the probability information at the time of the outcome, they could only choose between the options in the beginning of the next trial. We included only a reward probability, rather than also an effort probability, so as to not increase the cognitive demands of the task even further. Additionally, effort only having to be executed on some trials would have reduced the effort requirement; data from piloting and pre-screening of participants suggested that this might have led to some participants completely ignoring the effort dimension, which would have meant that we could not have assessed their behavioral or neural measures of effort learning.

After participants selected an option, it was highlighted until the ensuing outcome phase. In the outcome phase (Figure 1B), participants were first shown the reward and effort magnitudes of the option they had chosen, as well as whether they received a reward or not (in other words whether the outcome was a real secondary reinforcer indicating a specific monetary payment or instead hypothetical). If they received a reward, the current trial’s chosen reward magnitude was added to their total reward accumulated so far (Figure 1B, on the bottom of the screen). They were then shown the reward and effort magnitudes for the option they had not chosen. During the outcome phase, participants could thus use the displayed information to update their estimates of the reward and effort magnitudes associated with the choices. While they were shown whether they received a real reward or not, this should be irrelevant for their future choices as the informational content was the same. This is because how likely any choice was to be rewarded only depended on the probabilities that were explicitly cued at the time of choice and these probabilities changed randomly from trial to trial. In other words, whether a choice was likely to lead to a real or a hypothetical reward was not a feature of the choice that was sustained across trials or which differentiated it from the other choice and participants were made aware of this.

Finally, independently of whether the reward was real or hypothetical, participants had to perform the effort phase of the trial (Figure 1C). Participants had to exert a sustained effort by selecting circles that appeared on the screen using the trackball mouse. The circles were added to random positions on the screen in threes every three seconds (up to a total equal to the chosen effort magnitude). To make the task more effortful a random jitter (5 pixels, total screen size was 1280x800 pixels) was added to the mouse movement and circles only had a 70% probability of disappearing when clicked on. Furthermore, we pre-screened participants and only invited participants for the fMRI session if they had perceived the effort as aversive and were willing to trade-off money to reduce the effort that they needed to exert.

Participants had 25s to complete the clicking phase and otherwise lost money equivalent to the potential reward magnitude of the chosen option (participants failed to complete the effort phase on less than 1% of trials). Thus, the amount that could be lost was also independent of whether the reward had been real or hypothetical.

On most trials (100 out of 120) participants had to chose between the two options with changing reward and effort magnitudes. The reward magnitudes were set between 0 and 20 pence and the effort magnitudes were set between 0 and 15 circles that needed to be clicked. On the remaining trials (‘Special-option-trials’, SOTs), participants had to choose between one of the changing options and one of two fixed options whose values participants learned in a training session outside the scanner. The value of both fixed options was 7.5 pence, but one had a fixed effort magnitude of 4 circles and the other had one of 12 circles. The SOTs were included to ensure participants learned the values of each choice, rather than just their preference for one option over the other (a relative preference for one option over the other would not enable participants to choose effectively on the SOTs).

Participants performed 120 trials of the learning task inside the scanner and an additional 120 trials afterwards on the next day outside the scanner to increase the number of trials for the behavioral data analysis. Each participant performed the same two schedules in randomized order.

Interspersed with the 120 learning trials, there were 20 trials on which participants just had to indicate which option had a higher mean effort magnitude. These trials were included to ensure participants paid attention to the effort dimension. They were not given feedback about their choice. These trials were not included in the data analysis.

**Training.** Participants were informed about the features of the task in two training session before the scan, including the fixed number of trials they would perform. This ensured that they did not perceive low effort options as having a potentially higher monetary value because taking them might allow participants to move on to the next trial more quickly and to perform more trials with more chances to win money. Further details of the training were as follows: In the first training session (45min), participants performed a version of the task without a learning component, i.e. not only the probability, but also reward and effort magnitudes were explicitly shown. This training ensured that participants were familiar with the features of the task, for example, that they understood what the probability information meant. We also used this session to exclude participants before the fMRI session that did not find the effort sufficiently aversive to produce robust effects on behavior. In a second training session (1h), we instructed participants about the learning task that they later performed in the fMRI scanner. At the end of the training, participants were queried about how they made decisions (specifically, they were asked “What are you thinking about when you’re making your decision”). All participants reported trying to learn the reward and effort magnitudes and using the explicitly displayed probabilities to make decisions. No participants reported using information about whether reward had been real or hypothetical on the last trial when making choices on the next trial. This suggested that participants were well aware of how to do the task before the beginning of the scan.

**Experiment timings:** The options were displayed for 1.4 to 4.5s before participants could make a choice. After the choice was made, the chosen option was highlighted for 2.9 to 8.0s. Next, the outcome was first displayed for the chosen option (1.9-2.1s), then for the unchosen option (1.9-6.9s). Participants then performed the effort exertion task (0-25s). Finally, the trial ended with an ITI (2.3-7.5s). The delays used ensured that we could examine the brain activity in the different phases of the task separately. There were no confounds between the brain activations in decision, outcome or effort exertion phase. All durations were drawn from Poisson-like distributions to increase design efficiency by having some long delays.

**3) Bayesian model used for behavioral and fMRI analysis**

The prediction and prediction error regressors in the behavioral and fMRI analyses were obtained by applying a standard Bayesian learning model to the data (similar to the one described in Behrens et al. [5]). In short, the Bayesian model estimated the current reward/effort magnitude predictions based on the previous trials for each of the four magnitudes (option A reward magnitude, option A effort magnitude, option B reward magnitude, option B effort magnitude) separately. It did this by taking into account the following properties of the experimental task: 1) The reward/effort magnitude outcomes were determined by separate underlying reward/effort distributions for each magnitude (normal distributions). 2) The mean of a magnitude distribution could sometimes change. 3) How quickly each mean changed could vary over the course of the experiment. Sometimes, the mean changed more quickly (high volatility) whereas at other times, it changes more slowly (low volatility). 4) The volatility of an attribute was not static but could also change over the course of the experiment. In other words, the parameters that the model estimated on every trial for each attribute were the reward/effort magnitude prediction (mean and variance), the volatility, and the volatility change.

The model estimated these parameters for the current trial based on last trial’s parameter likelihoods and the last trial’s attribute outcomes. It did this using Bayes’ rule, which is the most efficient way for updating beliefs given new evidence. In contrast to the fitted models, this model did not have any free parameters. For details on the specific mathematical implementation of the Bayesian learner, please refer to Behrens et al. [5]. The only noteworthy change compared to Behrens et al. (2007) was that whereas they tried to predict binary outcomes using a beta-distribution we wanted to predict numerical outcomes using a normal distribution and thus added an additional parameter estimating the width of the normal distribution for the magnitudes.

That the model captured the behavior of our participants well can be seen in Figure 2: Reward and effort magnitudes derived from the Bayesian model emerged as highly significant predictors of participants’ choices when choices are either binned according to these magnitudes (Figure 2A) or when these magnitudes are included as predictors in a regression analysis predicting choices (Figure 2B). Furthermore, model comparison (Figure 2C) showed that a model that uses these Bayesian reward and effort magnitude estimates (‘Bayesian – Add’) provided the best fit to the data.

**4) Behavioral supplementary regression analyses**

**Learning differences independent of interfering factors**

In regression analysis bGLM4, we assessed whether citalopram affected the use of PEs, independently of interfering factors. The regression predicted whether participants ‘stayed’ with the previous trial’s option or ‘switched’ to the alternative. As main factors of interest, we included relative (in favor of the ‘stay’ minus in favor of the ‘switch’ option) RPEs and EPEs. As regressors of no interest, we included the relative reward probabilities (displayed to participants on each trial), the relative reward and effort predictions and the last trial’s reward type (real vs. hypothetical).

$$Y_{bGLM4}=\beta_{0} +\beta_{1}{RPE}_{t-1}+\beta_{2}{EPE}_{t-1}+\beta_{3}{RewardProbability}_{t}+\beta_{4}{RewardMagnitudePrediction}_{t-1}+\beta_{5}{EffortMagnitudePrediction}_{t-1}+{\beta_{6}RewardType}_{t-1}$$

Analysis bGLM5 assessed whether effort learning was interfered by reward learning (analogous to analysis bGLM3b). We included as two regressors of interest relative EPEs separately on the quartile of trials with most favorable and the quartile of trials with most unfavorable RPEs. As regressors of no interest we also included the relative EPEs in the remaining trials (i.e. the half of trials when relative RPE was neither high nor low), the relative explicitly shown probabilities, the relative reward and effort predictions, the relative effort prediction errors and the reward type. We compared the resulting regression weights for EPEs using t-tests.

**Effort exertion.**We also analyzed whether, related to previous findings in animals [6], citalopram would affect the exertion of effort per se and particularly the motivating effect of irrelevant reward information on the exertion of effort. In the regression analysis (eGLM1), we used the clicking rate on each trial as the dependent variable and as regressors of interest the magnitudes of the rewards of the two options (the one that was chosen and the one that was not chosen), as well as reward type (real vs. hypothetical reward). Was also included as additional regressors the chosen option’s effort magnitude (i.e. the number of targets participants had to click) and the unchosen option’s effort magnitude. The regression was run using MATLAB’s glmfit, with a log link function as the clicking rate data were constrained to be larger than 0. In this analysis, we only included data from the behavioral session outside the scanner as the EPI sequence sometimes affected the reliability of the trackball mouse during the effort exertion phase during the scan.

$$Y_{eGLM1}=\beta_{0}+\beta_{1}{EffMagOutcome}_{chosen}+\beta_{2}{EffMagOutcome}_{unchosen} +\beta_{3}{RewMagOutcome}_{chosen}+\beta_{4}{RewMagOutcome}_{unchosen}+\beta_{5}RewardType$$

**5) MRI – additional details for data acquisition, pre-processing and ROI selection**

**Data acquisition.** Structural MRI and fMRI measurements were taken using a Siemens 3 Tesla MRI scanner. For the fMRI, we used a Deichmann echo-planar imaging (EPI) sequence[7] [time to repeat (TR): 3s; 3x3x3mm voxel size; echo time (TE): 30ms; flip angle: 87°; slice angle of 15° with local z-shimming] to minimize signal distortions in orbitofrontal brain areas]. This entailed orienting the field-of-view at approximately 30° with respect to the AC-PC line. We acquired between 1100 and 1300 volumes (depending on the time needed to complete the task) of 45 slices per participant. Additionally for each participant, anatomical images were acquired with a T1- weighted MP-RAGE sequence, using a GRAPPA acceleration factor of 2 (TR: 2200ms; TE: 4.53ms; inversion time: 900ms; voxel size: 1x1x1 mm on a 176x192x192 grid) [same protocol as [8]].

**FSL analyses.** We used the standard settings in the FMRIB’s Software Library (FSL) [9] for image pre-processing and analysis. Motion was corrected using the FSL tool MCFLIRT [10]. This also provided the six motion regressors used in the whole brain analysis (fGLM1). Functional images acquired were first spatially smoothed (Gaussian kernel with 5mm full-width half-maximum) and temporally high-pass filtered (3 dB cut-off of 100s). Afterward, the functional data were manually denoised using probabilistic independent component analysis [11], visually identifying and regressing out obvious noise components [12]; we considered only the first 40 components of each participant which had the greatest impact to interfere with task data (total up to 550). We note that the results shown in the paper remain the same when no ICA components are removed (denoising) or when slice time correction is applied to the data. We used the Brain Extraction Tool (BET) from FSL [13] on the high-resolution structural MRI images to separate brain matter from non-brain matter. The resulting images guided registration of functional images in Montreal Neurological Institute (MNI)-space using non-linear registrations as implemented in FNIRT [14]. The data was pre-whitened before analysis to account for temporal autocorrelations [15]. Statistical analysis was performed at two levels. At the first level, we used an event-related general linear model (GLM) approach for each participant. On the second level, we used FMRIB’s Local Analysis of Mixed Effects (FLAME 1) [16] with outlier de-weighting and tested the single group average. The main effect images are all cluster-corrected results with the standard voxel inclusion threshold of z=2.3 and a standard cluster significance threshold p<0.05. We also analyzed data in regions of interest (ROIs), extracted from spheres with a 3 voxel radius, identified in MNI standard space on the basis of orthogonal whole group analyses.

**6) Correlations between neural and behavioral prediction errors**

The aim of this analysis was to assess whether neural and behavioral prediction errors correlated with each other. For this, we included for each individual participant their regression weight (from analysis bGLM4) for relative (in favor of the ‘stay’ or the ‘switch’ option) effort (fGLM3a) or reward (fGLM3b) prediction error at the group level in FSL. The analyses were otherwise set-up exactly like fGLM1. We tested for correlations (at whole-brain corrected level p<0.05) between the relative behavioral EPE/RPE and the most closely corresponding neural regressor, i.e. the relative (contrast level difference between the regressor for the option that was chosen minus regressor for the option that was unchosen) EPE/RPE.

**Supporting information references**

1. First MB (1996) User's guide for the structured clinical interview for DSM-IV axis I disorders: SCID-I: Biometrics Research Department, New York State Psychiatric Institute.

2. Scholl J, Kolling N, Nelissen N, Wittmann MK, Harmer CJ, et al. (2015) The Good, the Bad, and the Irrelevant: Neural Mechanisms of Learning Real and Hypothetical Rewards and Effort. J Neurosci 35: 11233-11251.

3. Vetencourt JFM, Sale A, Viegi A, Baroncelli L, De Pasquale R, et al. (2008) The antidepressant fluoxetine restores plasticity in the adult visual cortex. Science 320: 385-388.

4. Palminteri S, Clair AH, Mallet L, Pessiglione M (2012) Similar improvement of reward and punishment learning by serotonin reuptake inhibitors in obsessive-compulsive disorder. Biol Psychiatry 72: 244-250.

5. Behrens TE, Woolrich MW, Walton ME, Rushworth MF (2007) Learning the value of information in an uncertain world. Nat Neurosci 10: 1214-1221.

6. Liu Z, Zhou J, Li Y, Hu F, Lu Y, et al. (2014) Dorsal raphe neurons signal reward through 5-HT and glutamate. Neuron 81: 1360-1374.

7. Deichmann R, Gottfried JA, Hutton C, Turner R (2003) Optimized EPI for fMRI studies of the orbitofrontal cortex. NeuroImage 19: 430-441.

8. Chau BK, Kolling N, Hunt LT, Walton ME, Rushworth MF (2014) A neural mechanism underlying failure of optimal choice with multiple alternatives. Nat Neurosci 17: 463-470.

9. Smith SM, Jenkinson M, Woolrich MW, Beckmann CF, Behrens TE, et al. (2004) Advances in functional and structural MR image analysis and implementation as FSL. Neuroimage 23 Suppl 1: S208-219.

10. Jenkinson M, Bannister P, Brady M, Smith S (2002) Improved Optimization for the Robust and Accurate Linear Registration and Motion Correction of Brain Images. NeuroImage 17: 825-841.

11. Beckmann CF, Smith SM (2004) Probabilistic independent component analysis for functional magnetic resonance imaging. IEEE Trans Med Imaging 23: 137-152.

12. Kelly RE, Jr., Alexopoulos GS, Wang Z, Gunning FM, Murphy CF, et al. (2010) Visual inspection of independent components: defining a procedure for artifact removal from fMRI data. J Neurosci Methods 189: 233-245.

13. Smith SM (2002) Fast robust automated brain extraction. Hum Brain Mapp 17: 143-155.

14. Jenkinson M, Beckmann CF, Behrens TE, Woolrich MW, Smith SM (2012) Fsl. Neuroimage 62: 782-790.

15. Woolrich MW, Ripley BD, Brady M, Smith SM (2001) Temporal autocorrelation in univariate linear modeling of FMRI data. Neuroimage 14: 1370-1386.

16. Beckmann CF, Jenkinson M, Smith SM (2003) General multilevel linear modeling for group analysis in FMRI. Neuroimage 20: 1052-1063.
